# Comprehensive genomic analysis of *Bacillus paralicheniformis* strain BP9, pan-genomic and genetic basis of biocontrol mechanism

**DOI:** 10.1016/j.csbj.2023.09.043

**Published:** 2023-10-03

**Authors:** Muhammad Asif, Zhang Li-Qun, Qingchao Zeng, Muhammad Atiq, Khalil Ahmad, Aqil Tariq, Nadhir Al-Ansari, Jochen Blom, Linda Fenske, Hissah Abdulrahman Alodaini, Ashraf Atef Hatamleh

**Affiliations:** a Department of Plant Pathology and MOA Key Lab of Pest Monitoring and Green Management, College of Plant Protection, China Agricultural University, Beijing 100193, China; b Beijing Advanced Innovation Center for Tree Breeding by Molecular Design, Beijing Forestry University, Beijing, China; c Department of Plant Pathology, University of Agriculture, Faisalabad 38000, Pakistan; d School of Ecological and Environmental Sciences, East China Normal University, Shanghai 200241, China; e Department of Wildlife, Fisheries, and Aquaculture, College of Forest Resources, Mississippi State, University, MS 39762-9690, USA; f Lulea University of Technology, Lulea 97187, Sweden; g Bioinformatics and Systems Biology, Justus Liebig University, Giessen 35392, Germany; h Department of Botany and Microbiology, College of Science, King Saud University, P.O. Box 2455, Riyadh 11451, Saudi Arabia

**Keywords:** *Bacillus paralichenformis*, Evolution, Pan genome, Core genome, Secondary metabolites, Peptides, HGT, Plant_bacteria interactions

## Abstract

Many *Bacillus* species are essential antibacterial agents, but their antibiosis potential still needs to be elucidated to its full extent. Here, we isolated a soil bacterium, BP9, which has significant antibiosis activity against fungal and bacterial pathogens. BP9 improved the growth of wheat seedlings via active colonization and demonstrated effective biofilm and swarming activity. BP9 sequenced genome contains 4282 genes with a mean G-C content of 45.94% of the whole genome. A single copy concatenated 802 core genes of 28 genomes, and their calculated average nucleotide identity (ANI) discriminated the strain BP9 from *Bacillus licheniformis* and classified it as *Bacillus paralicheniformis*. Furthermore, a comparative pan-genome analysis of 40 *B. paralicheniformis* strains suggested that the genetic repertoire of BP9 belongs to open-type genome species. A comparative analysis of a pan-genome dataset using the Kyoto Encyclopedia of Genes and Genomes (KEGG) and Cluster of Orthologous Gene groups (COG) revealed the diversity of secondary metabolic pathways, where BP9 distinguishes itself by exhibiting a greater prevalence of loci associated with the metabolism and transportation of organic and inorganic substances, carbohydrate and amino acid for effective inhabitation in diverse environments. The primary secondary metabolites and their genes involved in synthesizing bacillibactin, fencing, bacitracin, and lantibiotics were identified as acquired through a recent Horizontal gene transfer (HGT) event, which contributes to a significant part of the strain`s antimicrobial potential. Finally, we report some genes essential for plant-host interaction identified in BP9, which reduce spore germination and virulence of multiple fungal and bacterial species. The effective colonization, diverse predicted metabolic pathways and secondary metabolites (antibiotics) suggest testing the suitability of strain BP9 as a potential bio-preparation in agricultural fields.

## Graphical Abstract

**Figure:**
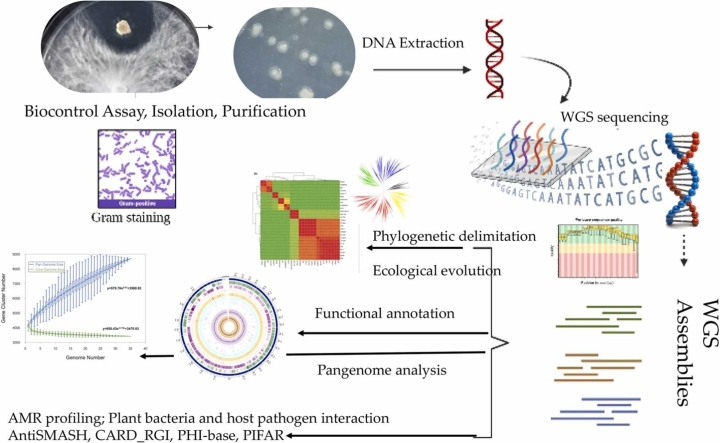

## 1 Introduction

*Bacillus* strains are a widespread clade of bacteria inhabiting diverse niches and could be isolated e.g., from soil, water, plant rhizosphere, mammals, and humans [1]. These species could be either deleterious or beneficial for their hosts. Most soil and plant-associated bacteria, especially *Bacillus* species, are commonly described as plant growth-promoting rhizobacteria (PGPR). They are renounced in producing various bioactive compounds, biologically fixing nitrogen, producing phosphatases, facilitating iron (Fe) intake, increasing the production of phytohormones, and ultimately causing induced systemic resistance [1], [2], [3]. Cohn and Koch (1872) established the genus *Bacillus*, and *Bacillus subtilis* has been implied as a model organism to study the genetic and developmental mechanisms in Gram-positive strains [4], [5], [6]. Application of certain members of *Bacillus* spp. can act as biofertilizers, as well as their competitive root colonization results in promoting seedling emergence and improving plant biomass [7], [8] by reducing disease pressure. They compete for nutrient resources, carbon, and iron and produce antibiotics and lytic enzymes [9], [10], [11], [12]. Thus, they are considered potential sources of pharmaceutical antibiotics, and such secondary metabolites play an essential role in bacteria fitness in the environment [13], [14], [15], [16].

*Bacillus licheniformis* and *B. paralicheniformis* are saprophytic, spore-forming, and facultative anaerobic species that are closely related but different from *B. subtilis* strains. The phylogenetic identification of these strains has always been challenging before the availability of complete genome sequences of *licheniformis* or *paralicheniformis* strains [17], [18], [19], [20]. The *B. subtilis* clade was further divided into *licheniformis* and *paralicheniformis* by identifying a novel strain, *B. paralicheniformis* KJ-16 [21]. Later, nine *B. paralicheniformis* strains were phylogenetically discriminated from 46 *B. licheniformis* strains [22], and the most conserved sequence of fencing (*fenB*) was proposed as a distinguishing marker [18]. *B. paralicheniformis* G-1, isolated from the deep sea, is a valuable source of enzymes degrading petroleum-based polystyrene compounds [23], and *B. licheniformis* strain SB3086 is a valuable bioformulation to inhibit many fungal pathogens [24]. Both strains are of equal importance; however, comparative genomic studies of *paralicheniformis* strains have received less attention than other *Bacillus* species. Besides the *B. paralicheniformis* KJ-16 [21], MDJK30 [22], Bac-84, and *B. licheniformis* strain SB3086, and ATCC 14580/DSM13 [25], no further strains exhibiting both antibacterial and plant growth-promoting activity have been documented.

Prior to the advent of extensive sequencing technologies, researchers in the fields of genetics and microbiology have utilized multi-locus sequence typing (MLST) and multi-locus sequence analysis (MLSA) as methods to investigate the systematics of various genera and species [26]. Furthermore, every newly sequenced strain revealed a remarkable genetic resemblance among members of *B. subtilis*, making it challenging to differentiate them using 16 S rRNA phylogeny. In addition to the 16 S rRNA, the utilization of housekeeping genes (gyrases *gyrA*, *gyrB*, and *rpoB*) that are conserved across multiple strains has proven valuable in elucidating population dynamics and the occurrence of evolutionary recombination. However, with increasing available sequences, all these methods started showing certain inherent limitations when constructing phylogenetic relationships. In order to achieve accurate lineage discrimination, it is necessary to have access to complete genome sequences, as only they provide all the necessary information to pinpoint the phylogenetic and evolutionary relationships among species. Now, pairwise ortholog comparison and synteny strategies have become prevalent in the investigation of core genomes, i.e., genes shared by all members of a selected set of genomes [27], [28], [29]. The concept of the pan-genome, as the totality of all unique genes in a group of genomes, was first described in S*treptococcus* species [30]. The pan-genome contains;.

• the core genome (genes present in all strains),
• the accessory genome (genes present in a subset of strains), and
• singleton genes (strain-specific genes).

Earlier, Hernández-González et al. [31] analyzed 79 representative *Bacillus* genomes and identified 196 core genes. Later, a comparative analysis of multiple genomes belonging to *Bacillus subtilis* and *cereus* clades was conducted by Nikolaidis et al. [32] and identified 114 core proteins as prominent fingerprint proteins, with only four proteins of known function as strict fingerprints. Pan-genome analysis has received particular attention for addressing genomic relatedness with much higher accuracy and confidence, projecting the evolutionary and phylogenetic distribution beyond niche specificity. With increasing available genome sequences, genome sequencing and assembly quality are becoming increasingly crucial as sequencing errors accumulate in pan-genome analyses. As an additional approach, the comparisons of the ANI between whole-genome and draft genome sequences have become a popular and widely accepted approach for delimiting species boundaries, usually with an implemented cut-off value of ∼96% identity [33], [34] with much higher accuracy and confidence.

There has not been much comparative genomic research conducted on *B. paraliceniformis* about closely related strains, and the genetic characteristics of these strains have been relatively understudied. There is a need for novel strains with effective antibiosis capabilities against numerous pathogens so that new genes could be explored to combat rapidly evolving interacting pathogens, which compelled researchers to explore other sources of genomic material and strains. This study isolated and identified a soil bacterium, BP9, from a lawn typically cultivated with ornamental plants. The bioinformatics analysis of sequenced strains can be utilized to establish a correlation, thereby enhancing comprehension of the interactions between microbial strains and their host plants. These insights allow us to evaluate the strain`s potential suitability as bio-formulation for agricultural applications.

## 2 Materials and Methods

### 2.1 Strains and media used

*B. paralicheniformis* BP9 was isolated from a small field usually cultivated with ornamental plants at China Agricultural University, Beijing, China, and stored in our laboratory (Biocontrol Key Laboratory of China Agricultural University). *E. coli* DH5α, *B. paralicheniformis* BP9, and *B. subtilis* 168 were cultured in LB medium at 37 °C while *Pseudomonas syringae* DC3000 at 28 °C. *Elsinoae* sp. (Grape anthracnose), *Phytophthora capsica, Botrytis cinerea, Rhizoctonia solani, Coniella* sp., and *Thielaviopsis* sp. were cultured in PDA plates at 25 °C. MSgg medium; 5 mM potassium phosphate 100 mM (pH 7), Mops 2 mM (pH 7), MgCl_2_ 700 μM CaCl_2_ 50 μM, MnCl_2_ 50 μM, FeCl_3_ 1 μM, ZnCl_2_ 2 μM thiamine 0.5%, glycerol 0.5%, glutamate 50 μgL^-1^, tryptophan/50 μg L^-1^, phenylalanine [35]. NBRIP medium (10 g/L dextrose; 2.5 g/L Ca_3_(PO_4_)_2_; 5 g/L MgCl_2_; 0.25 g/L MgSO_4_; 0.2 g/L KCl; 0.1 g/L, (NH_4_)_2_SO_4_; 1.5% agar) as described by [36] and incubated at 30 °C for 4 days. Whenever necessary, Appropriate antibiotics were added at the following concentrations: Ampicillin, 50 μg mL^-1^, Erythromycin 4 μg mL^-1^, Gentamicin, 30 μg mL^-1^, and Kanamycin, 50 μg mL^-1^.

### 2.2 Antibiosis assays against bacteria and fungi

The antibiosis activity of BP9 was assayed by following Weselowski et al., [37] with slight modifications. *B. paralicheniformis* BP9 was pre-cultured overnight in LB liquid medium. Hyphal plug of *Fusarium* sp. and 7 other fungal strains *Elsinoe* sp. (Grapevine anthracnose), *Phytophthora capsica*, *Botrytis cinerea*, *Rhizoctonia solani*, *Coniellla* sp. (white rot of grape), *Thielaviopsis* sp., and *Phytophthora sojae were* taken from a fresh culture plate and placed in the center of a PDA plate. Then, a 3 µL cell suspension of BP9 was spotted on both sides of the plug at a distance of 2 cm. Plates containing such co-culture strains were incubated for another 5 days at 28 °C. The antimicrobial activity was tested using Gram-positive and Gram-negative bacterial strains *B. subtilis* 168, *E. coli* DH5α, and *P. syringae* DC3000 and were seeded with fresh culture suspension, 1 mL of the respective suspension was seeded with 20 mL LB semisolid medium (1:20), dried in a clean chamber, followed by inoculation of BP9 in the center to measure the inhibition zones after three days of co-incubation or till optimal growth.

### 2.3 BP9 genome sequencing

DNA was extracted with the CTAB method [38]. The purity and concentration were analyzed using an Ultra-micro spectrophotometer (NanoDrop), followed by agarose gel electrophoresis. Then, the genomic DNA was sent to the commercial sequencing company Sinobiocore (China). For the sequencing of a sample named BP9, Illumina Hiseq 2500 with a 400 bp-insertion pair-end library platform was used. All reads were Quality Checked (QC) by a Trimmomatic tool to remove wrong insertion, primers, adapters, and poor regions. Subsequently, the filtered reads were *de novo* assembled by Edena (Exact DE Novo Assembler) [67] The bacterial genome sequencing of the assembled contigs was annotated by the PROKKA pipeline v1.1.1[39]. BLAST software aligned all CDS against databases nr, nt, EGGNOG, and KEGG for additional function mapping [31].

### 2.4 Qualitative analysis of PGPR characters

The universal method of Schwyn and Neilands [68] was followed to determine the qualitative siderophore production. Exopolysaccharide (EPS) production of isolates was measured by using the method [40]. For P-solubilization, NBRIP medium was used to culture the strain BP9 at 28 °C for four days. For oxidase activity, 1% Kovács oxidase reagent was utilized. The filter paper was dipped in this reagent. Bacterial cells were picked with a loop and placed on the filter paper. Isolates with oxidase activity changed the color of filter paper from light purple to dark purple [41]. To observe catalase activity, H_2_O_2_ drop was added on glass slides containing bacterial colony. If there were bubbling on slides, those isolates were positive for catalase activity [42]. In brief, BP9 was cultured in 3 mL LB liquid (200 rpm) at 37 °C to OD600 = 0.8, and cells were centrifuged to remove LB liquid at 6,000g for five min., followed by washing them with phosphate-buffered saline (PBS, 137 mM NaCl, 2.7 mM KCl, ten mM Na_2_HPO_4_, and two mM KH_2_PO_4_), and resuspended in 100 mL PBS. Swarming motility was tested in LB plates containing 0.7% agar-agar, first dried in a clean hood for 20 min, followed by spotting 3 µL BP9 cells suspension in each plate triplicate [43], [44], and kept at room temperature (5 h). Again, the plates were air-dried for 2 h on a clean bench and the diameter of the swarming zone.

BP9 biofilm and quantification assays were formed in MSgg medium using 12 healthy plates. As per good size, the optimized cell suspension of strain was mixed into 4 mL of MSgg medium, followed by static incubation at 28 °C for ∼48 h. Extra cells and the liquid were drained off slowly and carefully while biofilm in each well was washed thrice with 3 mL sterile saline, fixed with 2 mL of 99% (v/v) methanol for 15 min, followed by air-drying. The dried biofilms were stained with 2 mL of 1% crystal violet (CV) for 10 min. After standard washing of the stained biofilm, it was diluted 200 times, and a reading was taken at OD570 [35].

Wheat seedlings were grown as described by Ashraf et al. [40]. Wheat (JZ74) seeds were surface sterilized in 75% (v/v) ethanol thrice, followed by soaking in sodium hypochlorite solution (5% active chlorine) for 30 min and rinsed 8–10 times subsequent wash steps with sterile water for 2–4 min each for the removal of NaHCLO. Sanitized seeds were put into the petri dish (90 mm diameter) containing sterilized filter paper and soaked with 1.5 mL of sterilized water. The petri dish was incubated in moisture chambers at 28 °C for 48 h. Germinating seeds were soaked in 1.0 × 106 CFU/mL BP9 for control ddH_2_O. Later, Murashige and Skoog (MS) prepared as suggested by manufacturer instructions and added 0.8% disco-agar, autoclaved and dried. Seeds were transferred into these sterilized bottles containing 100 mL MS media. In addition, the germinated seeds were transplanted in plastic pots (70 mm × 75 mm) containing unsterilized vermiculite for pot experiments in the greenhouse. The bottles were kept in a tissue culture room, and pots were kept in greenhouse chambers (28 °C/20 °C day/night temperatures, 3500 Lux light for 16 h/d, and 70% relative humidity).

### 2.5 Phylogenetic analysis and genomic characterization

A phylogenetic tree for delineation of *B. paralicheniformis* BP9 was constructed with *Bacillus subtilis* lineages genomes, including six *licheniformis*, seven *paralicheniformis* strains, and *Paenbacillus polymyxa* M1 as an outgroup (Fig. 4). All sequences used for comparisons were downloaded from the National Center for Biotechnology Information (NCBI)—single-copy core genes as orthologs were acquired after aligning all protein orthologs from those 28 genomes. Alignment was done by a multiple alignment program for amino acid or nucleotide sequences (MAFFT v.7.310) [45], followed by a concatenation of protein sequences from a conserved block. Eight hundred-two genes were separated as single-copy core genes from multiple alignments of tested protein by Gblocks [46]. Single copy core genes were further implied in the PROTGAMMALGX model with 100 bootstrap replicates and RAxML v.82.10 with 1000 replicates for the maximum likelihood tree to construct. The ANI values for the heatmap describing the phylogeny of the 28 genomes were calculated by JSpecies, followed by MUMmer4x for protein alignments [47]. Further visualization was carried out in MEGA X [48]. ANI values-based heatmap was developed and visualized JSpecies. Housekeeping genes were aligned and visualized with molecular evolutionary genetics analysis MEGA version X. For initial phylogenetic testing. The NJ tree was also constructed using the latest MEGA version X [48]. BP9 and other strains previously reported [83] for comparing species phylogeny in Fig. 7 constructed. They visualized MEGA version X and the evolutionary analysis of five penguin proteins and five bacitracin proteins [48]. The COG and KEGG pathways analysis was performed as described [49], [50]. CRISPRCasFinder, CARD, Phast, alien hunter, and genomic islands were accessed using process [51], [52], [53], [54], [55]. MobileOG-db (Beatrix-1.6) was used to identify different genomic elements [56].

**Fig. 1:**
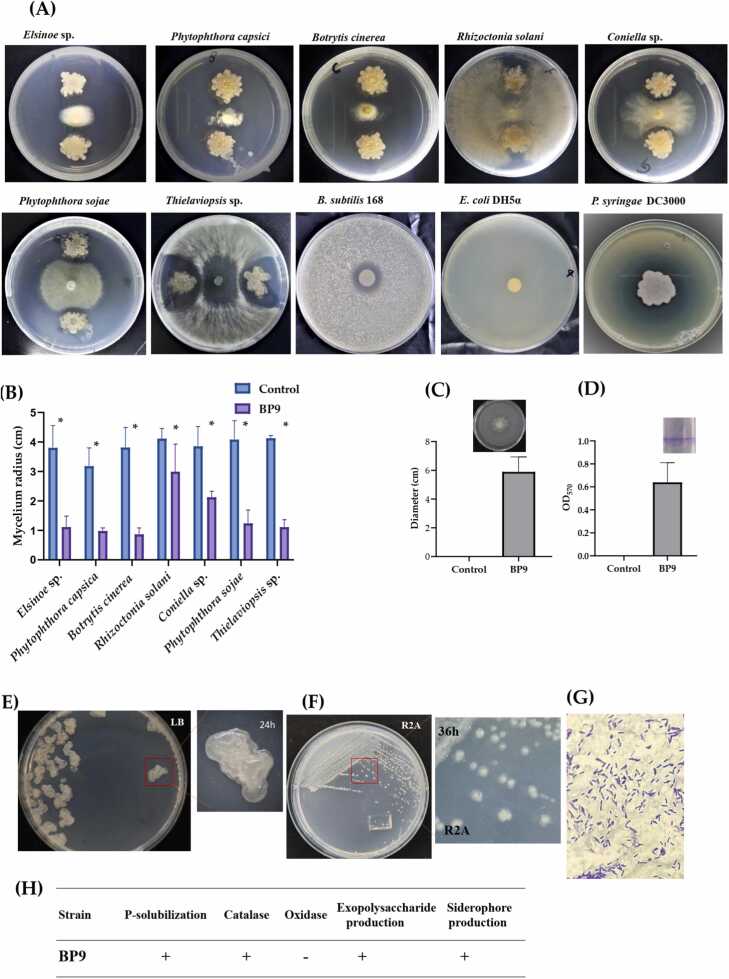
Antibiosis activity and morphological characters of BP9. **(A)** BP9 inhibited the growth of *Elsinoe* sp., *Phytophthora capsici*, *Botrytis cinerea*, *Rhizoctonia solani*, *Coniella* sp., *Phytophthora sojae*, and *Thielaviopsis* sp. Pathogens were placed in the center, and BP9 was inoculated side by side to test mycelial inhibition. (**B**) The measured mycelial radius in Control and inhibited by BP9. (**C)** BP9 cells were washed with respective buffer and spotted on LB plate for swarming activity screening by measuring the diameter. **(D**) The adhesion to abiotic surface of the BP9 in LB medium at 8 h was measured by the amount of crystal violet retained in the bacterial biomass regarded as biofilm-forming activity of BP9. **(E-F)** BP9 colony morphology on LB medium after 24 h at 37 °C and on R2A agar and their magnified view. (**G)** Gram staining of BP9. (**H)** PGPR characters like Phosphate-solubilization, oxidase, catalase, EPS and siderophore production.

**Fig. 2:**
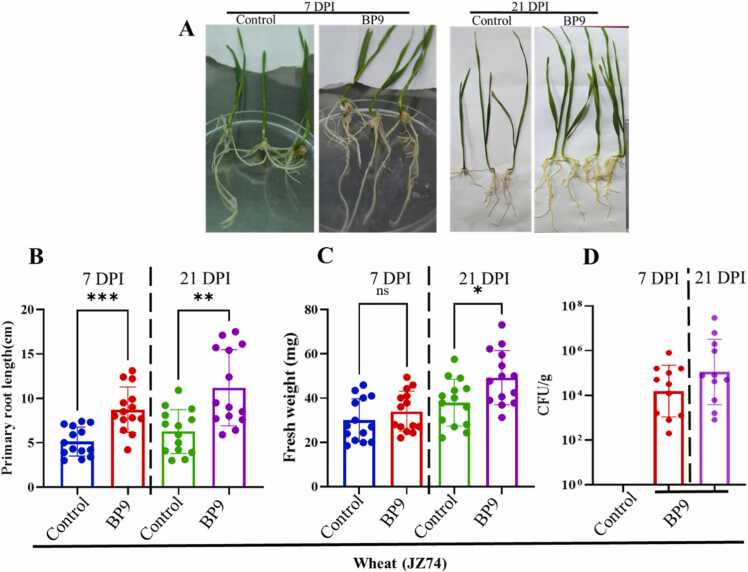
Effect of BP9 on root length, root fresh weight and colony forming units per gram (colonization) on wheat roots. (**A**) Wheat (JZ74) seedling grown for 7days and 21days show significant impact of BP9 on root length. (**B**) Measured root length in cm, (**C**) root fresh weight in mg, and (**D**) colonization of BP9 in colony forming units per gram of roots after 7- and 21-days post-inoculation (DPI). Data is the average of gnotobiotic and greenhouse experiments. Statistical significance was calculated using One-way ANOVA (non-parametric). Asterisk * indicates the level of significance at p < 0.05, and double or triple asterisks indicate the p < 0.01–0.001. For the sake of control, ddH_2_O water was used. The experiment was repeated in triplicate in MS media for wheat culture in control and in the greenhouse.

**Fig. 3:**
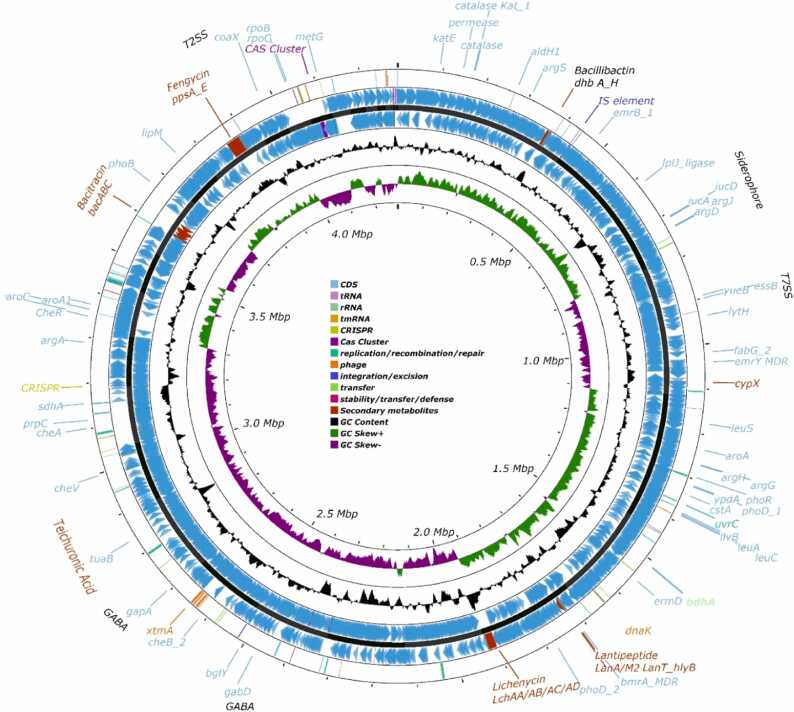
The circular representation of BP9 genome. Inner rings show the scale, GC skew, and GC content of the BP9 genome, from inside to outside, respectively. The next 3 rings indicate the forward and reverse strands together with the chromosome's backbone (contigs; Black ring). The outermost ring represents essential genes related to secondary metabolites (bacitracin, bacillibactin (siderophores), lantipeptide, fengyin (*fenABCD*), and lichenycin), genes of phages (*cypX*, *dnaK*, *xtmA*), IS elements, Type II, VII secretion system, catalases (*katA*, *katE*), permeases, ligases (*lipl*, *lipJ*), Beta galactosdase *bglY* and essential Plant factors (*bdhA*, teichuronic acid *tuaB*, GABA, *bmrA*, *emrY*, *phoR*, *phoD* (phosphatases) involved in plant-bacteria interactions.

**Fig. 4:**
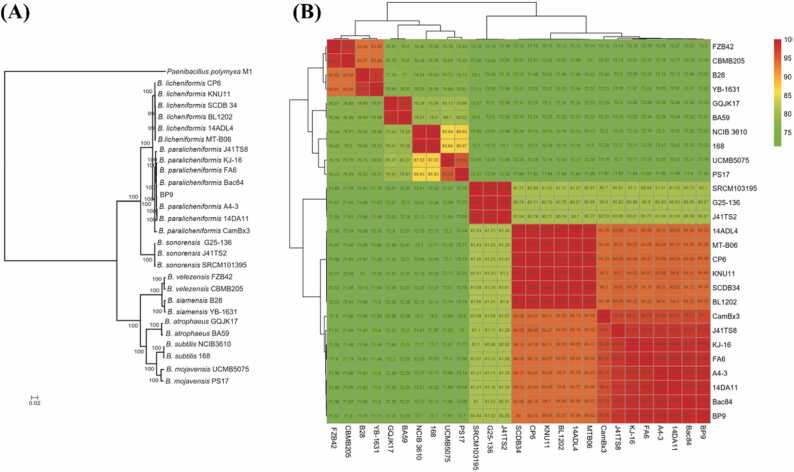
Phylogenomic analysis of *B. paralicheniformis* BP9. **(A)** A concatenated alignment of 802 single copy core proteins of 28 genomes of tested strains using *Paenibacillus polymyxa* M1 as outgroup was used to construct maximum likelihood phylogenetic tree with branch support values shown at the respective nodes. **(B)** A heatmap of the average nucleotide identity (ANI) of 27 strains for the discrimination of strains representing the same species with high values of reddish colors.

**Fig. 5:**
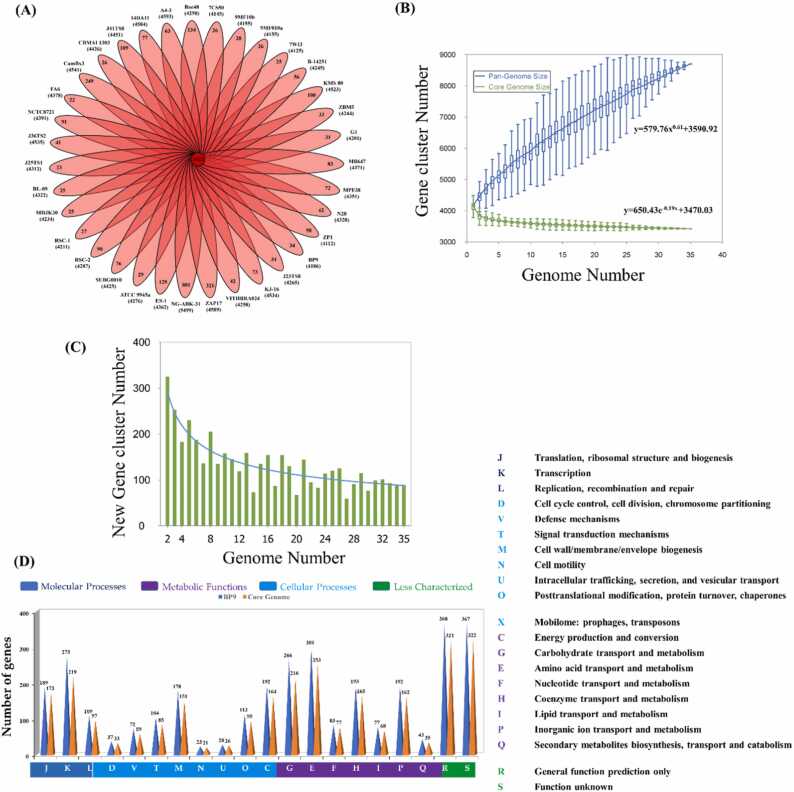
Pan genome of 40 *Bacillus paralicheniformis* strains and and COG analysis. (**A**), Pan-genome analysis of 40 *paralicheniformis* strains, including BP9 conducted by PGAP and visualized by PanGP. (**B)** Pan-genome and Core genome extrapolation curve of 40 tested strains, calculated by Heaps’ law formula y = κ*x*^*γ*^*+ b* for the pan genome and an exponential decay function model (y = A*e^Bx^ + K) for the core genome size in relation to the number of analyzes genomes. (**C**) Curve analysis using again an exponential decay function model for number of new genes as per each added new genome, suggesting more than 83 new gene families in the pangenome. (**D)** COG functional category analysis for comparing the metabolic potential of strain BP9 with the metabolic capabilities of 39 other tested strains.

**Fig. 6:**
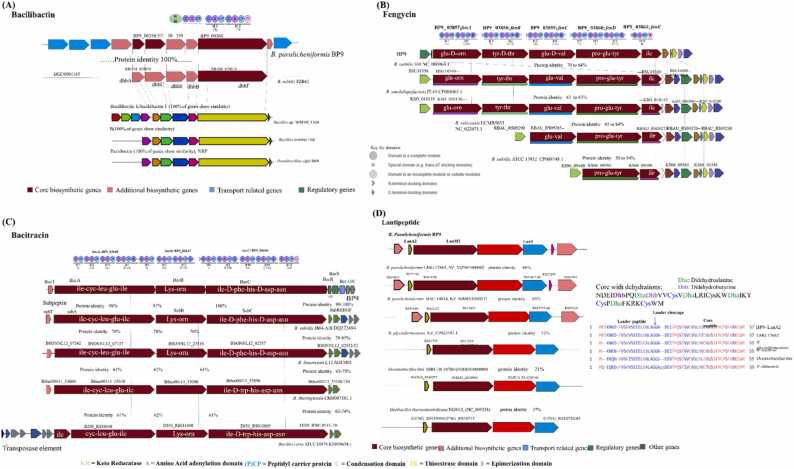
Genome mining of bacillibactin, fengycin, bacitracin, and paralichenicidin-like gene clusters. Genetic organization of gene clusters for bacillibactin (A), Fengycin **(B),** bacitracin (C) and lantipeptide (D). Same genes are shown in the same color and linked by dotted lines for similarity and identity. Percentage amino-acid identities of each peptide with bacitracin operon in *B. paralicheniformis,* Insertion sequence, biosynthetic genes, transport genes, regulatory genes and other genes are shown in black, red, blue, green and gray, respectively. The protein encoded domains and their substrates in biosynthetic genes shown at the top are predicted by antismash*.*

**Fig. 7:**
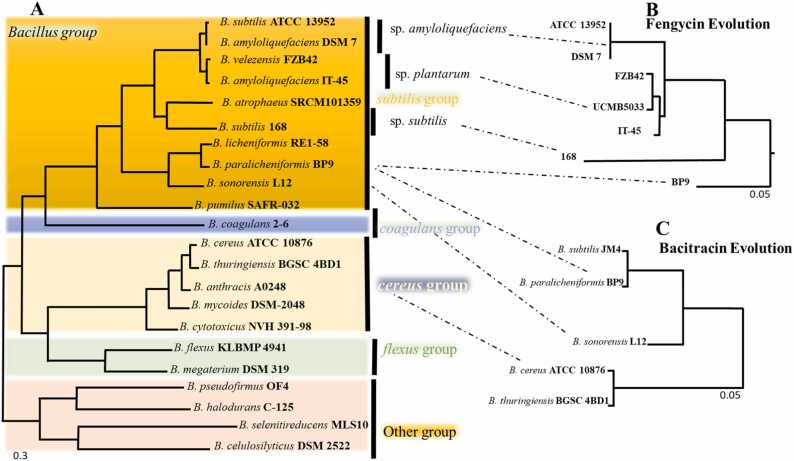
Phylogenetic tree of species, fengycin, and bacitracin using maximum likelihood (mL) (**A**) The groupings of phylogenetically linked species found in the Bacillus tree include the previously described subtilis, coagulans, cereus, flexus, and "other" groups [59] based on Maximum-likelihood (mL) including the BP9 strain, (**B**) Maximum-likelihood phylogenomic tree using five protein of fengycin (*ppsABCDE* BP9_03857, BP9_03858, BP9_03859, BP9_03860, BP9_03861) (**C**) and bacitracin cluster specific proteins (BP9_03648, BP9_03647, BP9_03646, BP9_03645, BP9_03644).

### 2.6 Pan Genome Analysis of paralicheniformis strains

*B. paralicheniformis* genomes used in the pan-genome analysis are listed in Table S3. Pan-genome analysis was carried out by following [17]. Briefly, strain-specific and core genes of *paralicheniformis* genomes were picked from a pan-genome analysis using the PGAP software [57]. o do so, 40 *B. paralicheniformis* mentioned in Table S3 were used to cluster functional orthologs based on Gene Family (GF) amino acid sequences in PGAP. Further, the protein identity range of 50% similarity followed by the same overlap range longest reads with a cut-off e value of 1e-5 was selected for the PGAP-based pipeline-based protein similarity method. Among the 40 strains tested, shared gene families were considered core genomes, while the total full complement of genes in genomes (n = 40) was regarded as the pan-genome. For Fig. 5AB, trajectories and the sizes of core and pan-genome for increasing genome numbers were developed by regression algorithms already used by [27], [58]. Furthermore, the curve fitting model and Heaps’ law equation were used. However, visual trajectories of the pan and core genome were made by PanGP software with default parameters [17], [27].

### 2.7 Prediction of plant-bacteria and Host-pathogen Interactional

Different web-based pipelines were used to predict the genes related to plant bacteria and host-pathogen interaction. For former interactions, the PIFAR web service was used [59] and literature-based manual searches. BGCs were predicted by the bacterial version of antismash software [60]. The Virulence Factor Database (VFDB) [61] and PHI-base.org [62] were used to identify the virulence factors and host-pathogen interactional genes in the BP9 genome, respectively.

### 2.8 Statistical analysis

One-way analysis of variance (ANOVA) was performed by the GraphPad Prism, and statistical significance was calculated at P ≤ 0.05 across all treatments. Bar graphs were generated by GraphPad Prism v9.

## 3 Results

### 3.1 Morphology and antibiosis activity of BP9

To explore the genetic diversity and evolution of *B. paralicheniformis* strain BP9, we performed whole genome sequencing, followed by an in-depth genetic analysis, antibiosis essays, and morphological characterization. BP9 significantly inhibited the growth of *Elsinore* sp. (grapevine anthracnose), *Phytophthora capsica*, *Botrytis cinerea*, and *Thielaviopsis* sp. (causing crown rot, root rot and black rot diseases in different hosts). This indicates that the strain has the potential to repress different fungal pathogens. In addition, BP9 significantly curbed the growth of *Pseudomonas syringae* DC3000, the pathogen causing bacterial wilt. Interestingly, *Bacillus subtilis* type strain 168 and *Escherichia coli* DH5α were unaffected during co-culture with BP9 (Fig. 1 A-B). It suggested that BP9 is an antibacterial and antifungal agent for pathogenic strains, significantly inhibiting their growth but harmless for non-pathogenic strains. Moreover, strain BP9 possesses significant biofilm and swarming potential (Fig. 1 C-D). Besides this, BP9 has different morphology on different media and is Gram stain positive (Fig. 1 E-F). Further tests regarding the PGPR characters indicated that the strain BP9 can produce catalase, exopolysaccharides, and siderophores (Fig. 1 G-H).

PGPR bacteria naturally colonize the plant roots and positively affect plant growth and physiology. In order to test the effect of BP9 on plant growth and physiology of wheat, the germinating seeds were soaked with BP9 and measured the root length root fresh weight after seven days post inoculation (DPI) and 21 DPI (Fig. 2 A). BP9 significantly improved the seedling growth, as their root length increased compared to the control (water). In addition, root fresh weight and the population abundance of BP9 per gram of roots were significantly improved after 7 and 21 DPI. The tested biofilm, swarming, and colonization potential of BP9 indicate that the strain could act as PGPR for wheat (Fig. 2 B-D). The antibiosis assays, morphological characteristics, and plant growth promotion assays indicated that the strain BP9 contains a lot of hidden genetic factors regarding its molecular mechanisms in disease suppression and improving plant growth.

### 3.2 Genome sequencing for phylogeny and characterization of BP9

To gain insight into genetic features involved in phytopathogen inhibition, the BP9 strain was sequenced by Illumina HiSeq and was submitted to the NCBI for genome assembly (WGS accession ID: JAQIHP01). The obtained genome assembly consists of 52 contigs (*de novo*) with 4186 coding DNA sequences (CDS) and 4282 genes. Among the predicted CDS, 2905 were assigned proper functional annotation, including 411 with a putative function, and 1273 were annotated as hypothetical proteins. The total length of the genome was 4286,372 bp, with a mean G-C content of 45.94%. In addition, 83 tRNA and 12 rRNA genes were predicted in the genome (Table 1, Fig. 3).

**Table 1:** The de novo-genome annotation features of BP9.

Annotated Genome Features	
Genome Length	4286,372 bp
GC Content	45.94%
Contigs	52
Gene	4282
CDS	4186
tRNA	83
rRNA	12

Different genes of (bacterio-)phages and transposable elements play essential roles in niche adaptation and evolution. To predict the prophages and other important alleles present in the genome, categorical replication/recombination/repair (43 genes), transfer (22), stability/transfer/defense (5), and 33 phages regions representing the integrative, transposable, and conjugative elements were identified (Table S4) through the mobile-db database [30] And displayed at the outermost ring (Fig. 3, Table S4). The identified phage-related genes protect multiple bacteriophages. The *clpP/clpX* gene uses the AGGAG motif to interact with bacteriophage lambda O protein to degrade it. In addition, *fun* helps regulate the lysogenic process and is associated with iron/sulfur biogenesis proteins and their maintenance during the lytic cycle (Table S4). Lexi is helpful in SOS regulation and can act as a transcriptional repressor. Other phage-associated chaperonin proteins, i.e., GroEL, GroS, Hfq, FtsH, and ClpB, were also identified. At the same time, phage gene NusA is regulatory during phage infections, and defense was accomplished through rex phage element (Table S4). Among the categories, 13 transferase genes were present for competence (Table S4). Transferases are involved in a myriad of reactions in the cell. Most of the phages identified belong to lysis/lysogeny, infection, structural, and regulatory, while elements were related to competence (Table S4). Comparison of identified genetic elements of BP9 with *Bacillus subtilis* 168 and *Bacillus licheniformis* DSM13 revealed three unique mobile elements which belong to replication/recombination/repair (ParB), integration/excision (Tnp2_) and transferase/competence (CinA-2) (Fig S1 A). Besides these, three CRISPR, three types of CAS fragments, and five genomic islands were identified by *in silico* analysis (Fig. 3). The presence of identified mobile genetic elements suggested the indication of horizontal gene transfer (HGT) events that occurred during strain evolution. In addition, the BP9 genome was screened for HGT events using alien_Hunter [63] v1.10, which revealed 120 locations of a variable number of genes indicated horizontally transferred from different sources during the evolutionary process (Table S11).

The genome-wide type strain pipeline is a robust source of strain identification using next-generation sequencing (NGS) data at Gctype [64]. The sequenced genome was submitted for strain identification and blast analysis. It revealed BP9 has 99.16%, 99.21%, 98.7%, and 98.7% identity to the 16S-rRNA gene with the strains *B. licheniformis* ATCC-14580, *B. sonorensis* KCTC 13918, *B. subtilis* subsp. *inaquosorum*, and *B. subtilis* subsp. *spizizenii*, respectively, while 100% identity with type strain *B. paralicheniformis* KJ-16 (Table S6). Digital DNA–DNA hybridization (dDDH) was determined online (http://ggdc.dsmz.de/distcalc2.php) using formula 2 of the genome-to-genome distance calculation (GGDC) [65]. Formula 2 of the GGDC is the only function appropriate to analyze draft genomes and further delineate the species level based on dDDH. The type strain genome server (TYGS) pipeline was used according to the default parameters [65], [66]. The threshold value over 70% indicates that the strains are similar. The obtained dDDH and ANI values are listed in Table S7. The BP9 genome showed a dDDH of 57% with DSM-13/ATCC-14580 genome, a value lower than the threshold, but 92.6% with type strain KJ-16, suggesting the BP9 strain belonged to the *paralicheniformis* group. The calculated ANI values of BP9 with KJ-16 using ANIb and FastANI also revealed 98.90% and 98.91% identity, respectively, further proving the results obtained from different sources for identification (Table S7).

A phylogenetic tree for delineation of *B. paralicheniformis* BP9 was constructed using the maximum likelihood (mL) method containing 28 *Bacillus* species, including six *B. licheniformis* and seven *B. paralicheniformis* strains with *Paenbacillus polymyxa* M1 as outgroup. The tested 28 strains with 802 single copy core genes indicated the BP9 clustered with *the B. paralicheniformis* group (Fig. 3 A). These results were consistent with the former results of dDDH and ANI, suggesting that BP9 belongs to *paralicheniformis* strains and is most similar to Bac 84, A4_3, and 14DA11 (Fig. 4A). However, the obtained phylogenetic analysis does not allow to distinguish the strains by their geographical origin. It reflected a mixed trend of strains isolated from a similar niche and clustered the genomes independent from the habitat-specific grouping in tested strains (Fig. 4 A). Furthermore, we calculated the average nucleotide identity (ANI) values of different strains using JSpecies [33] to confirm the findings from phylogenetic analysis. The results revealed two distinct groups of selected *B. licheniformis* and *B. paralicheniformis* strains indicated in red, showing 100% identity with their respective group. Based on ANI data, *Bacillus subtilis*, and other strains were also separated into distinct clades as indicated by the heatmap (Fig. 4 B), and strains with ANI values > 95% are considered the same species [33]. The closest ANI values of both types of strains indicate that most regions of genomes are relatively conserved among intragroup of species. The close ANI values between *B. licheniformis* and *B. paralicheniformis* strains show that most regions of these genomes are relatively conserved. Furthermore, the matrix shows that *B. sororensis* relates more to the lichen group than to the rest of the Bacillus clade. All other species are clustered correctly according to the phylogeny. To get a broader overview, an additional mL tree of 167 *Bacillus* strains (42 *B. licheniformis*, 108 *B. paralicheniformis*, and 17 other *Bacillus* strains) was constructed based on 662 concatenated single copy core genes using FastTree2 [63] via EDGAR3.0 [67] to pinpoint the sister clade of BP9, which turned out to be *B. paralicheniformis* B4121 (Fig. S7).

### 3.3 Pangenome analysis B. paralicheniformis strains

The genetic diversity of BP9 was explored by comparing the pan-genome of 40 *B. paralicheniformis* strains, including BP9, using PGAP with translated CDS sets considering the orthologue clusters and strain-specific CDS in the total gene content. Among the 8700 protein-coding genes, 39.34% (3422) were present in all included genomes. The genome NG-ABK-31 sourced from the soil had the most significant number of distinct families, totaling 801 out of 5499 coding sequences (CDS). In contrast, the genome ZAP17 sourced from a hot spring had the second-largest number of unique families, amounting to 321 out of 4589 CDS. The genome FA6 sourced from the gut of grass carp had the lowest number of unique families, with only 22 detected among 4378 CDS (Fig. 5 A). Pan-genome versus core genome sizes of the tested strains were plotted against the number of total genomes (Fig. 5 A). The number of novel genes added with each new genome noticeably shapes the pan-genome curve, suggesting substantial differences in gene content among tested genomes.

Heaps’ law formula y = *κx*^*γ*^ + b was used to approximate the pan genome growth of *B. paralicheniformis.* An exponent *γ > 0* indicates an open pan-genome among species*.* For *B. paralicheniformis,* the resulting nonlinear model y = 579.76x^0.61^ + 3590.92 (Fig. 5 B) shows a quick growth of the pan genome and suggests substantial differences in gene content and frequent addition of novel gene families among the included genomes. The exponent γ > 0 indicates an open pan-genome among species. Consequently, the pan genome curve of 40 genomes also reflected that adding more new strains will still not fully describe the complete genetic repertoire of *B. paralicheniformis.* Any addition of novel strains will add up to the gene pool of the species due to the open pan genome.

Moreover, the relationship between genome number and new gene size was analyzed by curve fitting in mathematical model y = A*e^Bx^ + K, which suggests that the addition of each new genome could result in an average of more than 83 new genes in the pangenome, while y refers to the pan genome size, x refers to the number of sequenced genomes tested and A, B are fitting parameters calculated as 391.3 and − 0.42, respectively. Further, the power`s law exponential decay function model prediction resulted in 3179 core genes of *B. paralicheniformis* strains (Fig. 5 B).

Moreover, the relationship between genome number and new gene size was analyzed by curve fitting using a mathematical model of the number of accessory genes with the exponential decay function y = A*e^Bx^ + K, which suggests that the addition of each new genome could result in an average of more than 83 new genes in the pan-genome. Here y refers to the pan-genome size, x refers to the number of sequenced genomes tested and A, B and K are fitting parameters calculated as 650.43, − 0.19x, and accumulative of more than 83 new genes respectively. Further, the exponential decay function model prediction of the core genome size resulted in 3470.03 core genes of *B. paralicheniformis* strains (Fig. 5 B-C).

The COG assignment was utilized to analyze the genes of BP9. The results indicated that a substantial portion of these genes were assigned to the E category (9.3%, related to amino acid transport and metabolism), G category (8.28%, related to carbohydrate transport and metabolism), and K category (8.56%, related to transcription). These categories were consistently higher than in the pan-genome set from all other species or those observed in the core genome (Fig. 5 D). However, other categories were shown to be consistent with closely related strains, including translation and ribosomal structure, transcription, cell wall and membrane biogenesis, coenzyme transport and metabolism, and inorganic transport and metabolism. Nevertheless, there are substantial genes whose putative functions have yet to be determined, called hypothetical proteins or with yet unknown functions. To accurately identify their exact functions, more *in silico* investigations are required, particularly in the categories R and S. Both BP9 and the core genome exhibit significant functional constancy, regardless of their environment or geographic isolation point, extending beyond their interaction with plants. The predicted secondary metabolism genes and pathways allowed us to propose that most genes are conserved and associated with adaptation processes and competitive advantage in diverse habitats.

Furthermore, a considerable proportion of proteins have activities that remain unidentified, necessitating the use of functional genomics and computational analytic techniques to elucidate their possible roles. Subsequently, KEGG pathways associated with each category were visualized as a bar graph, depicting the gene count for each category (Fig. S2). The investigation revealed that the number of genes remained relatively stable and uniform across all examined strains in all COG categories and KEGG pathways when comparing the core genome of BP9 to the tested strains for the pan-genome (Fig. 5D, Fig. S2).

Comparative genomics and phylogenetic analysis among both lineages (para lichens) provide essential information about genetic relatedness and their ecological and evolutionary differences. For instance, the genome of *B. paralicheniformis* BP9 and the type strain *B. licheniformis* DSM-13 show minor differences in the Fastlane, which reflects the genetic closeness. Although both lineages are genetically similar, specific genomic fragments, including insertion sequence, prophages, and described secondary metabolic synthetases, are unique in *B. paralicheniformis* strains (Fig. 3). Such genes allow better adaption of *B. paralicheniformis* to grow in a variety of environmental niches relative to *licheniformis* strains. The genetic similarities followed by phylogeny may overlap in both lineages but not for species-specific habitats.

### 3.4 Analysis of antifungal metabolites of BP9

The analysis of genomes from individual paralicheniformis strains and licheniformis revealed distinct gene clusters associated with secondary metabolic synthases (Fig. S3). Anti-smash analysis suggested the presence of bacillibactin, bacteriocin, lasso peptide, lichenin, siderophore, terpene, CDPS, and fencing/pepstatin biosynthetic gene clusters (BGCs) present in *paralicheniformis*. Few of the *licheniformis* strains also have similar types of BGCs. Different genes of clusters encoding mentioned (BGCs were prevalent in both types of strains, but some strains were deprived of it, probably due to HGT or gene loss events that occurred during the evolution (Fig. S6, Table S8). Several strains were subjected to antismash analysis from both lineages and compared to the BGCs (Fig. S3). Most bacteria subjected to antismash in both lineages include genes that encode bacillibactin. Intriguingly, it was observed that genes encoding the BGC for bacitracin production were only found in one strain (SL-16) from the lineage *licheniformis*. In contrast, other members, including type strain DSM13, did not contain this cluster. Nevertheless, BGCs responsible for bacitracin production were present and prevalent in most *paralicheniformis* members, including BP9. These findings also suggest that HGT events have led to the loss of some genes within these BGCs, particularly the loss of the bacitracin gene in the *lichensformis* group (Fig. S3). Subsequently, BP9 harbors bacilli batin, bacitracin, fencing, and pentapeptide antibiotic gene clusters, along with four genes (*srfAABCD*) of lichenin (Table S8).

#### 3.4.1 Bacillibactin;

During antagonistic assays, Pst-DC3000 was significantly inhibited by certain antibiotics of BP9 secreted in agar plates (Fig. 1). Genomic analysis revealed a BGC for bacillibactin, a siderophore-based secondary metabolite as reported in *B. amyloliquefaciens* MBI600 and characterized to strongly inhibit the *P. syringae* DC3000 pathogen (Fig. 1). Manual blast analysis indicated that the gene biosynthesizing the bacillibactin is highly similar with MBI600 and additionally shows 100% protein identity to well-known strains *Bacillus subtilis* FZB42, *Bacillus* sp. WMMC1349, *Bacillus subtilis* 168, *Paenibacillus elgii* B69, and *B. amyloliquefaciens* IT-45 during antismash and PSI-blast analysis (Fig. 6 A). Bacillibactin is a class of siderophore compounds produced by *the B. licheniformis* group of strains, and the cluster is rigorously transferred through HGT events during evolution (Fig S6 A). The biosynthetic pathway of 2, 3-dihydroxybenzoate (dhb) can produce bacillibactin via a series of reactions catalyzed by a multienzyme complex. This complex is formed by the dimerization of *dhbACEBF* operons, which are present in the BP9 genome. The expected class of bacillibactin, as shown in Table S8, contains E and F. Further studies are needed to determine the precise quantity and specific composition of bacillibactin generated in BP9 and elucidate its genetic potential and structural characteristics. These investigations provide an exciting prospect for future study, particularly in evaluating its possible applications in bio-formulations.

#### 3.4.2 Fengycin

Alternatively, Plipastatin, specifically active against filamentous fungi, is biosynthesized by fengycin five synthetases genes encoded by NRPSs FenA-FenE (fenA-E) [43], [44]. The BP9 genome contains a single fegycin cluster *fenABCBCDE* with 86% protein identity to *the B. subtilis* FZB42 strain. The domains and orientation of genes encoding its proteins involved in fengycin biosynthesis in BP9 show striking similarity with *B. subtilis* 168, *B. amyloliquefaciens* IT-45, and *B. velezensis* UCMB5033 having identical gene orientations with a variable number of genes present in their genomes (Fig. 6 B). This suggests that strains shared a common ancestor during evolution and genetic elements likely *fen A-C* are interchangeable or transposable in different species, though they belong to core biosynthesis genes. The presence of fenA-C in different strains, either one or all, indicated that they had been transferred due to the transposition ability or HGT shifts. Manual protein blast analysis indicated the protein identity among intragroup was 98–100%, but inter-group identity varied with a minor similarity of 70% (Fig. S4). All the data support that the cluster biosynthesizing the fengycin was acquired as an HGT event from *a subtilis* ancestor (Fig. 6 B, Fig. S4).

Surprisingly, *B. velezensis* UCMB5033 and *B. subtilis* ATCC 13952 harbor only FenCDE and FenDE genes but no fenAB(C), suggesting an incomplete gene cluster (Fig. 6 B). This incompleteness of the biosynthetic genes *fenA-C* and part of *fenD* is also evident in the strains ATCC 13952 and DSM-7, which are non-plant-associated [13], [68]. It is well established that the complete fencing cluster is primarily present in plant-associated strains and can cause induced systemic resistance in tomatoes and beans [69], [70], suggesting that horizontal gene transfer events might have taken place from non-plant-associated strains (ATCC 13952 and DSM-7) and *fenAB(C)*, either of these was lost in evolutionary pressure in some strains (Fig. 6 B). Moreover, we did a fencing cluster-specific evolutionary analysis, which suggested the horizontal transfer of fencing among *the subtilis* group (Fig. 7 A-B) and then consistent transfer in later species (*licheniformis* and *paralicheniformis*), as also indicated by earlier researchers [68], [71]. BP9 actively inhibited the growth of different fungal and bacterial pathogens (i.e., DC3000), which supports the hypothesis that fencing-producing strains have the potential to inhibit the mycelial growth of multiple fungi associated with causing disease in plants.

#### 3.4.3 Bacitracin

is a common antibiotic used to suppress variable pathogens and is used for ophthalmic and skin ointments and probiotics in the poultry industry. Our anti-smash analysis found that the *B. paralicheniformis* BP9 contains all the essential genes to produce bacitracin, including three synthetase (*bacABC*) genes. Additional biosynthetic and transport-related genes, likely *bacT*, and *bacR*, *bacS* (two-component system), *bacE* (ABC transporter), and two additional genes (*bacGF*), were present in BP9 (Fig. 6 C). Manual protein blast analysis indicated that *B. sonoresis* L12 and *B. subtilis* JM4 showed the most striking resemblance and highest protein identity in gene *bacABC* synthetases domains (Fig. 5, C). In addition, *Bacillus sp.* TYF-LIM-B05, *Bacillus* sp. CPSM8, *Bacillus* sp. MSP5.4 and *Bacillus Haynes* NRRL B-41327 also contain *bacABC* genes with a protein identity of 95–100% (PSI and manual blast analysis). Besides these*, B. paralicheniformis* KMS-80, BL-09, LMG 17662, ATCC 9945 A, and 14DA11 contain the most similar loci (protein identity: 100%) identified during antismash and manual PSI-blast analysis (Fig. S5). Previously, the prevalence of bacitracin synthase genes among *B. licheniformis* strains depicted noticeable variations, and only one contained the bac operon (Fig S4). Only strain *B. licheniformis* S-16 retained a complete bacitracin operon, but other *licheniformis* strains, including the type strain DSM-13, contain no such whole operon (Fig S3). In addition, most of the *paralicheniformis* strains, along with BP9, harbor the bac operon, which suggests that the genes for bacitracin cluster adoption are HGT events during evolutionary pressure (Fig. S5, S6 B). The *bacABC* synthetase proteins of *B. paralicheniformis* BP9 were strikingly similar to another *B. subtilis* JM4 strain with a gene involved in the bacitracin cluster called subreption [72], [73]. Both strains' bacitracin synthetase BacABC and subpeptin subABC share 97–100% protein identity (Fig. 6 C). Nevertheless, most *B. subtilis* strains contain no subpeptin genes, similar to JM4, and the acquisition of the associated gene cluster is proposed to be taken from *B. paralicheniformis* via a recent single HGT event, which could be associated with *B. cereus* ATCC 10876 due an identified transposable element ahead of *bacT* gene (Fig. 6 C, Fig. 7 A/C). Uncovering the evolutionary history among intragroup species requires further studies of gene transfer and loss events. The bacitracin synthetase genes bacABC showed certain deviations based on different strains, but that sequence divergence does not affect the conserved bac domains' substrates (Fig. 6 C). The first two domains are pretty similar, but remarkably, the amino acids of the second A domain in back could be modified phe or d-trp, likely (ile-cys-leu-glu-ile-lys-orn-ile-phe-his-asp-asn and ile-cys-leu-glu-ile-lys-orn-ile-d-trp-his-asp-asn) in other *Bacillus* strains (Fig. 6 C). This difference might hint at the biological variances in bacitracin production in different strains, and even similar strains can produce multiple types of bacitracin depending on substrate availability and conditions. Other identified genes, *bacT*, *bacRS*, and *bacEFG* of different strains, encode for the two-component systems' transcriptional regulators and sensory proteins. These are primarily conserved in these strains and play a role in bacitracin self-resistance for the strains producing it (Fig. 6 C).

#### 3.4.4 Lantipeptides

Lantipeptides are considered a potential antimicrobial strategic weapon against multiple pathogenic bacteria. BP9 has one type of lantipeptide similar to LMG 17662 and Bac14814, with 86% and 85% protein identity (Fig. 6 D). LanA peptide is consistently transferred from *B. glycinifermentans* and *Oceanobacillus limi IBRC-M 14814* strains with significant protein identity of core and leader peptide (Fig. 5 E). Another protein, LanM (lanthionine), was 50% identical to the geobacillin class II lantibiotic biosynthetic gene cluster and belonged to the peptide of *Geobacillus thermodenitrificans*. For class-I lantibiotics, dehydration, and cyclization are carried out by two different enzymes, generically called LanB and LanC, identified in other species, whereas for class II lantibiotics, both reactions are performed by a bifunctional enzyme (LanM [74] as identified in BP9.

The secondary metabolites are somehow transferred as part or whole and considered an HGT event (Fig. S6). For instance, a part of the bacillibactin (the cluster) is being acquired (Fig. S6 A). Similarly, *ppsC* and *ppsD* (*fenC* and *fenD*) of fengycin cluster and *bacABC* synthetases of bacitracin and lantipetide clusters are horizontally transferred (Fig. S6 C, D). Both lineages contain different secondary metabolite gene clusters, and antismash analysis was conducted to find the exact cluster present (Fig. S3). It also indicates that some clusters are exchanged during evolutionary pressure (Fig. S3).

### 3.5 Identification of Genes Involved in host-pathogen and Plant Interaction

**Fig. 8:**
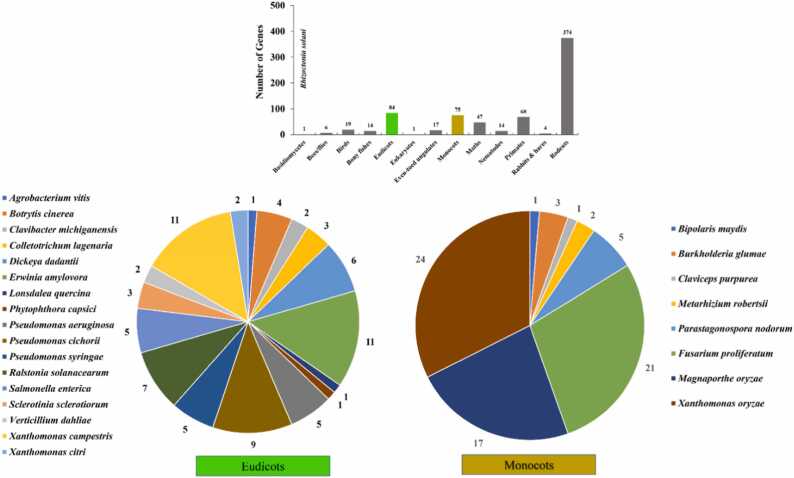
Multiple genes identified in BP9 provide resistance against plant pathogens potentially susceptible based on putative antibiotic pathways. The total number of tolerant genes in different categories is shown in the bar graph (Top). The number of genes associated with resistance against pathogens is displayed at the top of the pie chart, while number of disease-causing pathogens is displayed alongside the pie chart (Bottom).

In order to identify the gene involved in interaction with pathogenic fungi or bacteria, an *in silico* blast analysis of the BP9 genome using the PHI-base.org database [41] was carried out. The PHI-base blast results revealed 724 genes interacting with variable fungal or bacterial strains, including some tested in Fig. 1. BP9 is necessary for host-pathogen interaction in monocot and eudicot plant hosts. Seventy-five genes for host-pathogen interaction were identified in BP9 regarding monocot hosts, while 85 genes were found for the eudicots. BP9 can use these proteins to interact, resulting in reduced virulence or spore germination with the respective pathogens (Fig. 8). In monocots, relevant pathogens like *Parastagonospora nodorum, Fusarium proliferatum, Magnaporthe oryzae, and Xanthomonas oryzae* could be influenced by those 75 specific genetic determinants (proteins) in BP9. Among eudicots hosts, important pathogens like *Botrytis cinerea, P. aeruginosa, P. syringae, Salmonella enterica, Dickeya dadantii, Ralstonia solanacearum, Pseudomonas victoria, Erwinia amylovora, and Xanthomonas campestris* will be affected resulting in reduced spore germination and virulence during interaction with BP9 (Fig. 8). These plant host interaction (PHI) proteins could particularly impede the virulence and pathogenicity. However, identified genes (proteins) for primates and rodent-related pathogens indicate that a probiotic effect might also exist against several human pathogens (Fig. 8).

The genetic determinants encoding proteins in BP9 could reduce the virulence of bacterial and fungal pathogens during interaction (Table 2). *Elsinore* sp., *Phytophthora capsica*, *Botrytis cinerea*, *Rhizoctonia solani*, *Coniella* sp., *Phytophthora sojae*, and *Thielaviopsis* sp. are known to cause root rot or crown rot disease, as well as black or white rot. When tested during dual culture assays, BP9 significantly inhibited them. It is important to note that spore formation is crucial for several pathogens in a competitive environment. BP9 possessed predicted proteins capable of reducing the virulence (respective pathogen) and affecting the spore formation of the mentioned pathogens (Table 2). Most importantly, the *P. syringae* strain could affect the plants by causing bacterial leaf blight and speck blight. In addition to siderophore and secondary metabolites (bacitracin, fengycin), BP9 might use those predicted proteins (BP9_01400, BP9_03461, BP9_04050, BP9_01404, etc.) to interact with the *P. syringae* that could result in reduced virulence (Table 2). Overall, more genes are associated with bacterial leaf blight/bacterial blight and fewer with crown rots and wilts in different plant hosts.

**Table 2:** The pathogen-host interactions genes of the BP9 genome predicted by PHI-base web blast analysis.

**Gene**	**E/value**	**Disease**	**Exper. Host**	**Site**	**Prior penetration**	**Spore germination**
**BP9_01400**	2.02E-^131^	Bacterial leaf blight	*Oryza sativa*	leaf	Reduced virulence	Reduced
BP9_03461	2.11E-^97^	Black rot disease	*Raphanus sativus*	leaf	Reduced virulence	
**BP9_01402**	4.50E-^142^	Bacterial leaf blight	*Oryza sativa*	leaf	Reduced virulence	Reduced
BP9_04050	4.69E-^77^	Black rot disease	*Raphanus sativus*	leaf	Reduced virulence	
BP9_02361	2.53E-^63^	Rice blast	*Triticum*	leaf	Reduced virulence	Wild type
BP9_03158	9.70E-^89^	Bacterial wilt	*Solanum lycopersicum*	-	Lethal	
BP9_01180	6.03E-^147^	Rice blast	*Oryza sativa*		Reduced virulence	
**BP9_01509**	1.00E-^141^	Bacterial leaf blight	*Oryza sativa*	leaf	Reduced virulence	Reduced
**BP9_01404**	6.20E-^71^	Bacterial leaf blight	*Oryza sativa*	leaf	Reduced virulence	Reduced
BP9_01417	5.37E-^168^	Black rot disease	*Raphanus sativus*	leaf	Reduced virulence	
BP9_03672	4.20E-^169^	Grey mold	*Vitis vinifera*	fruit	Reduced virulence	
BP9_00148	6.24E-^179^	Fire blight	*Pyrus communis*	fruit	Reduced virulence	Wild type
BP9_03027	8.87E-^72^	Crown infections, leaf spot, and foliar blight	*Capsicum annuum*	leaf	Effector (plant avirulence determinant)	
BP9_04033	2.83E-^57^	Citrus canker	*Citrus sinensis*	leaf	Reduced virulence	
BP9_03387	1.08E-^60^	Bacterial leaf blight	*Oryza sativa*	leaf	Reduced virulence	Reduced
BP9_03188	3.34E-^62^	Bacterial blight	*Oryza sativa*		Increased virulence (hypervirulence)	
BP9_02039	6.62E-130	Black rot disease	*Raphanus sativus*	leaf	Reduced virulence	
BP9_03412	2.58E-^37^	Black rot disease	*Raphanus sativus*	leaf	Reduced virulence	
BP9_00337	1.72E-^136^	Infections	*Lactuca sativa*	leaf	Unaffected pathogenicity	Reduced; aberrant
BP9_00272	1.45E-^123^	Black rot disease	*Brassica rapa*	leaf	Reduced virulence	
BP9_02679	8.77E-^112^	Bacterial leaf blight	*Oryza sativa*	leaf	Reduced virulence	Reduced
BP9_02468	5.33E-^143^	*Stagonospora nodorum* blotch	*Triticum aestivum*	leaf; seedling	Reduced virulence	Wild type
BP9_03541	1.16E-^137^	Grey mold	*Vitis vinifera*	fruit	Reduced virulence	
BP9_00301	1.07E-^124^	Infections	*Lactuca sativa*	leaf	Unaffected pathogenicity	Reduced; aberrant
BP9_02678	9.51E-^117^	Bacterial leaf blight	*Oryza sativa*	leaf	Reduced virulence	Reduced
BP9_02785	3.38E-^44^	Poplar bark cankers	*Populus canadensis*	bark	Reduced virulence	
BP9_00801	3.40E-^137^	Bacterial speck of tomato	*Arabidopsis thaliana*	leaf	Reduced virulence	Wild type
BP9_00624	6.70E-^44^	Grey mold and rot disease	*Solanum lycopersicum*	leaf	Reduced virulence	
BP9_04186	3.90E-^123^	Crown gall of grapes	*Vitis vinifera*	shoot	Loss of pathogenicity	

The additional web-based tool, “PIFAR” [59], was used to identify different genetic factors involved in plant-bacteria interactions. Rhizosphere competence is linked to the capability to form biofilms. The genome of BP9 contains the complete set of genes implicated in biofilm, some exopolysaccharides, and lipo-polysaccharides identified by the “PIFAR” tool (Table S4), as tested in Fig. 1. BP9 has similar unique genes encoding for a collagen-related GXT structural motif and are probably involved in surface adhesion or biofilm formation as identified in *B. amyloliquefaciens* FZB42 [44]. The summary of genes relevant to plant-bacteria interactions is given in Table S9. BGCs related to bacillibactin, lanthipeptide, bacitracin, fengycin, fusaricidin, and fosfomycin and their resistance-conferring genes were identified (Table S9). Moreover, cell wall degrading enzymes, cobalt-zinc-cadmium resistance, and resistance or detoxification resistance for penicillin, fluoroquinolones, and fosfomycin-producing genes are predicted (Table S9). Additionally, BP9 contains one beta-lactamase, beta-galactosidase, chromium compounds and multidrug resistance efflux pump, isothiocyanate resistance, *sapABCDF*, *katB*, *dps* (Table S9, S10). Besides biofilm and adhesion, chemotaxis (CheABCDVYW) and plant hormones regarded as microbial- or pathogen-associated molecular patterns MAMPs were identified in BP9. Moreover, the factors associated with metabolism include A-tryptophan/trpCG, glutamate synthase *gltBD*, *aroK*, *aroQ*, *asnB*, aroC, and purine biosynthesis *purD*. In addition, some putative genes producing volatile compounds acetoin and 2,3-butanediol are identified that could act to induce systemic resistance and plant biostimulant (Table S9).

## 4 Discussion

This study underpins the antibiosis activity of *B. paralicheniformis* BP9 invitro against fungi and bacteria. The significant antimicrobial activity against different fungal pathogens, especially *Conellia sp.*, *Phytophthora,* and *Botrytis* sp., indicates that the BP9 contains diverse secondary metabolites to suppress pathogenic fungi of the different phyla. The Gram-negative *P. syringae* (Pst-DC3000) was also inhibited, suggesting that BP9 is equally effective against bacterial pathogens of different types and its potential application in disease suppression. Some tested PGPR characters, including siderophore, EPS, protease casein degradation, and catalase, were consistent with previous studies [22], indicating the significant ability of *the paralicheniformis* strain to produce multiple compounds during the interaction, which is also necessary for its adaptation. Besides the strain BP9, *B. paraliceniformis* Bac84 was also reported to possess peptides capable of inhibiting *Pseudomonas syringae*, *Staphylococcus* sp, *Clostridium* sp., *Listeria* sp., and *Salmonella* sp. strains [75], [76].

The number of sequenced genomes could allow one to understand phylogenetic and functional differences among different bacteria. 16 S rRNA and MLSA with conserved alleles in the genome [58], [77], [78] could sufficiently help delineate species levels. However, the more accurate the phylogenetic reconstruction, the more genetic data like genome sequence to separate groups. The inter-species phylogenetic cohesion of a group such as Bacillus was examined using 802 concatenated core genes. This analysis allowed for the discrimination between *B. licheniformis* and *B. paralicheniformis*, ultimately classifying the BP9 into the *paralicheniformis* group. In addition, the *silicon* investigations suggest that the BP9 strain has undergone explicit evolutionary adaptations under selective pressures. Although the phylogenetic analysis does not separate the strains based on habitat, the identified exchanged fragments (genes) during adaptation indicate their co-evolution with plants, plant-pathogenic bacteria, and fungi [79]. The unique phage-related genes, IS elements, transposases, lyases, phosphatases, and Type II and VII (Esat/EssB) secretion systems in BP9 reflect the effective adaptation in various niches and discriminate it from other strains (Fig. 5B). The presence of the plant peptides class of compounds contributes to the significant antagonistic activity of such strains for such a range of pathogenic microbes [74], [80], [81], [82]. Siderophore biosynthesis makes the BP9 strain competitive in iron-scarce conditions, improving the strain's fitness capacity. The identified genes of phages help transmit antibiotic resistance and virulence genes by inserting their genome into the host DNA, hence playing a significant role in bacterial evolution [56]. They may also become lysogens by reproducing themself and killing their temporary host organism. Besides the phages, another term referred to as genetic competence is state of the art, permitting the uptake of DNA prevalent in both Gram-positive and Gram-negative bacteria [43]. *B. subtilis* 168 can take up a wide range of DNA fragments due to the extensive selection process in the laboratory, while the other strains have the attenuated ability. This process is strikingly conserved and mediated by core genes (ComEC et al.). These genes are proposed to interact with competence proteins, which are also explicitly conserved proteins RecA, SsbB, and Smf [83]. This gives us strong hints to understand the extent of genetic competence, which explains genetic variability by gene acquisition, at what frequency it occurs, and which signals trigger it at what specific environmental conditions [43], [84], [85].

Moreover, the analysis of BP9 with other strains, the pan-genome analysis, shows an asymptotic trend in the core development curve and, at the same time, an open pan-genome. This indicates that novel genomes might be required to describe the core genetic repertoire of *B. paralicheniformi*, while the general genetic repertoire is unlimited due to frequent HGT events [86]. The ortholog gene families were consistent regarding the metabolic pathways involved in energy generation and amino acids transport to the core genome of other *B. paralicheniformis* strains [87]. The COG [50] and KEGG pathways [49] analysis highlighted that the BP9 single genome harbors more genes related to the three categories E, G, and K. Categorically, these genes are involved in amino acids and carbohydrate metabolic systems (central metabolism), and pivotal in strain`s adaptation in the rhizosphere [7], [87]. This implies that the bacteria originated from the soil and their close relative in plants, and their products show a consistent collinearity. Such diverse metabolic activity, secretion systems, detoxification, and stress response systems could reduce genetic fitness costs to adapt to the specific nutrient environment effectively and enable PGPR strain to be used in agricultural fields.

The pan-core-based ortholog clustering of the protein families provides insights into unique genes coding functional protein sequences in the BP9 genome and the shared ancestry of various genomic elements and their evolutionary relationships. Analysis of pan-genome and single copy core genes, including *licheniformis* and *paralicheniformis,* could serve as a paradigm for plant-associated bacteria, which enabled us to discriminate BP9 from *licheniformis* strains and others. Previous reports indicate that most strains have > 8% of the genome devoted to antibiotic clusters, while in *Bacillus* species, 5% is dedicated to secondary metabolites production [81]. Surprisingly, the genome of BP9 contains BGCs of bacillibactin, bacitracin, fengycin, and antibiotic lantipeptide (lantibiotic_paralichencidin), which are rarely produced in *B. subtilis* strains, rather than lantibiotics mersacidin and subtilin, which indicate a significant portion of genome allocated for the production of these antibiotics [70].

Plant-associated rhizobacteria residing in specific niches undergo multiple HGT events (gene insertion, deletions), and the rhizosphere micro-environment and plants significantly influence these events. Besides the pan-genome analysis, the origination of BGCs and their distribution, including BP9, discriminated the unique features of both lineages and highlighted their cohesion and transformations in rhizosphere adaptation [69], [83], [86]. We observed significant deviations in the GC content of studied BGCs (Fig S1B, Fig S6), and the orientations and genetic scheme of gene clusters in different organisms are somehow coherent, suggesting the elements acquired by the HGT process during evolution, especially the gene cluster for fengycin and Bacitracin biosynthesis. The genetic structure and orientation might suggest sharing a common ancestor because of their interchangeable genetic elements (Fig. 6). Our results aligned with previous studies indicating the bacitracin cluster is consistently evolved and present in *B. cereus* and *subtilis* groups. However, flexus and *coagulans* are deficient in them [22], [24], [68]. Some transposable elements identified in ATCC 10876 support the acquisition of the bacitracin operon due to that flanking IS elements [88], [89] and distinguish the origin from *subtilis* order describing the ancestor's *Bacillus cereus*, *coagulans* through HGT.

Sequencing new strains can lead researchers to predict how different metabolic activities in similar bacteria are regulated and reveal the genetic basis for improving such metabolic activities [89], [90]. In particular, hypothetical proteins (HPs) are a new source of novel proteins useful for providing resilience against abiotic and biotic stresses. Genome sequencing of as many strains of the *paralicheniformis-licheniformis* group will make it possible to comprehensively compare these secondary metabolic synthases and HPs in both lineages, thereby offering new perspectives and strategies for facilitating future applications in agriculture and industry. Understanding the genetic basis of secondary metabolites will significantly assist future attempts to improve their applicability resulting in new molecular compounds assisting in proving the solution for antibiotic resistance.

PHI blast analysis of the BP9 genome could provide a basic framework to further explore the interaction of predicted genes against pathogens in reducing their virulence. It provides the basis for designing functional genetics screens and gene expression profiling of a strain to provide experimental support linking the functional relevance of the rhizosphere's cataloged genetic repertoire of plant-beneficial traits. Apart from phylogenetic studies, core genome studies co-relating the ecological and geographical characteristics assessment, functional genes associated with comparative distributions, can reveal the functional profile. *Whole genome sequencing techniques* are a relatively cheap and rapid way of addressing inter-species diversity and assist in determining the roles of the genes underlying distinct taxonomic ranks. It will enable the scientific community to conduct a deep ecological and evolutionary study with precise genetic markers [84], [91].

## 5 Conclusions

The rhizosphere strain BP9 contains the essential antibiosis potential invitro to combat fungal and bacterial pathogens. BP9 improved seedling vigor and plant growth due to its possession of PGPR characters. Additionally, the identified mobile elements, phage-associated genes, HGT-acquired secondary metabolites, and diverse metabolic pathways enabled the BP9 to utilize different energy sources for metabolism. Secondary metabolites, especially fengycin and bacillibactin (siderophore-based), pentapeptide, and bacitracin were the primary antibiotic weapons acquired through recent HGT events necessary for competitive interaction. The dDDH, ANI, and concatenated genes of 28 genomes strongly differentiate the phylogenetic relationship of BP9 with *licheniformis* and *paralicheniformis*. *In-silico* pangenome analysis of *paralicheniformis strains* suggested that they belong to an open pan-genome genetic repertoire, but potential genetic diversity requires substantial experimental evidence. The identified genes regarding multiple plant-bacteria interactions and host_pathogen interaction in BP9 suggest futuristic studies about multiple plant fungal pathogens, resulting in reduced sporulation. The detailed phylogenetic analyses identifying striking differences in secondary metabolites, their protein domains, and substrates offer a new perspective suggesting novel research directions concerning genetic and transcriptomic studies to unveil their mechanism.

## Funding

The authors extend their appreciation to the Researchers supporting project number (RSP2023R479) King Saud University, Riyadh, Saudi Arabia. This work also was supported by the National Natural Science Foundation of China (31901845, 31872020).

## CRediT authorship contribution statement

**Muhammad Asif:** Conceptualization, Writing – original draft preparation, Methodology, Project administration, Software, Formal analysis. **Zhang Li-Quan**: Supervision, Validation. **Qingchao Zeng:** Software, Writing – review & editing. **Muhammad Atiq**: Writing – review & editing. **Khalil Ahmad**: Writing – review & editing. **Aqil Tariq**: Supervision, Writing – review & editing. **Nadhir Al-Ansari**: Funding, Writing – review & editing. **Jochen Blom**: Writing – review & editing. **Linda Fenske**: Writing – review & editing. **Hissah Abdulrahman Alodaini**: Writing – review & editing. **Ashraf Atef Hatamleh**: Writing – review & editing. All authors have read and agreed to the submission of the current version of the manuscript.

## Declaration of Competing Interest

The authors declare no conflict of interest.
