# Supplementary material for: Comprehensive genomic analysis of Bacillus paralicheniformis strain BP9, pan-genomic and genetic basis of biocontrol mechanism
Source: Comput Struct Biotechnol J. 2023 Oct 3;21:4647–62. doi: 10.1016/j.csbj.2023.09.043 (PMC10568305; doi:10.1016/j.csbj.2023.09.043)
Supplement: Supplementary file 1 — Supplementary material [file mmc1.docx]

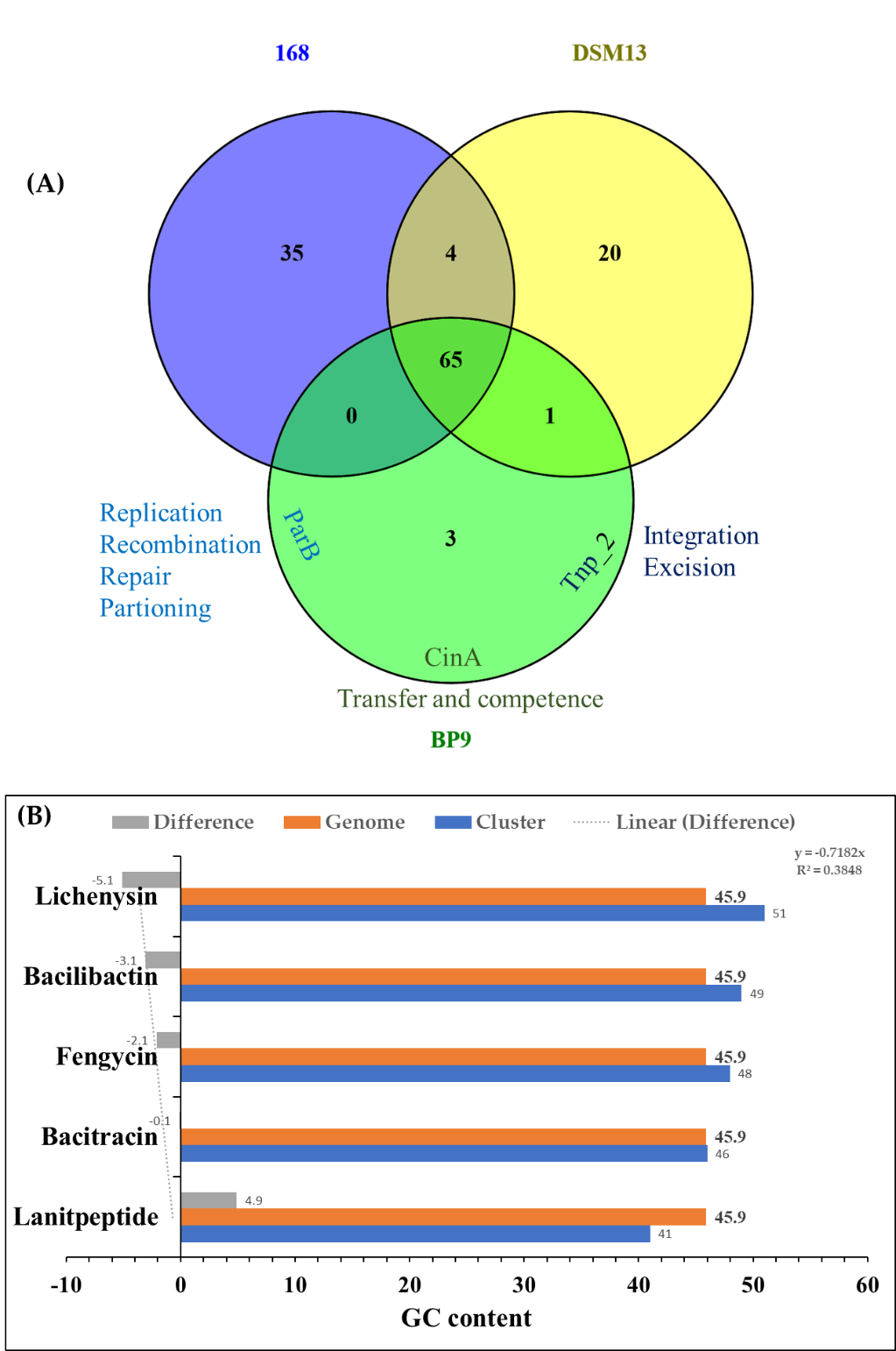


**Figure S1. (A)** Comparative analysis of BP9 mobile elements (phages) with *Bacillus subtilis* 168, *Bacillus licheniformis* DSM13**;** BP9 contain 3 unique phage elements **(B)** GC content of BP9 secondary metabolites and its difference from gene clusters.


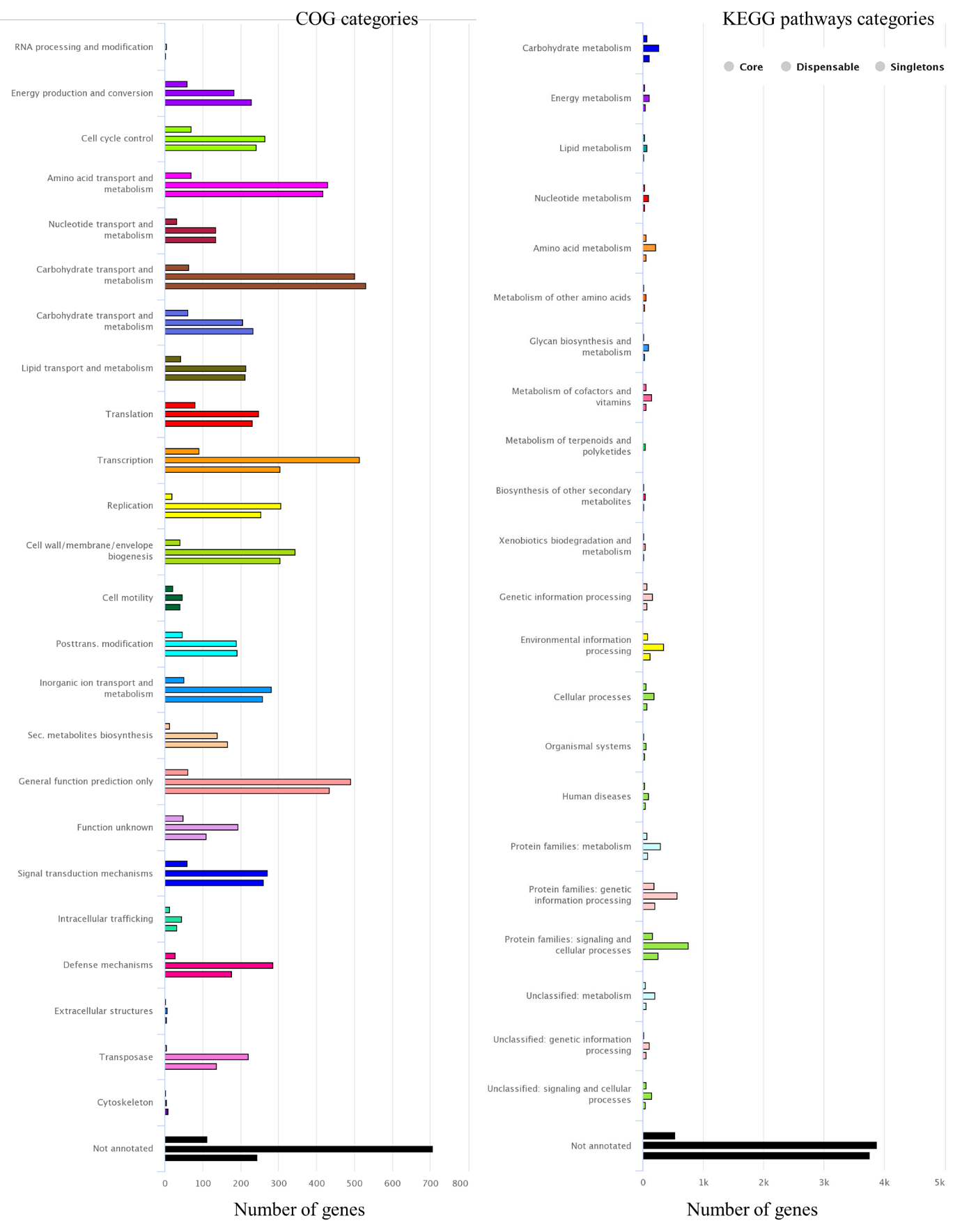


**Figure S2;** COG and KEGG pathway analysis of BP9 with other *B. paralicheniformis* strains describing core dispensable and singletons (pangenome). First bar indicates core, the next dispensable and the last singletons in each category. More number of genes are associated to not annotated genes

**Figure S3.** Comparative secondary metabolites analysis of *B. licheniformis* and *paralicheniformis* strains. Red color indicates the presence, while blue represents the absence of that cluster in the respective strains.


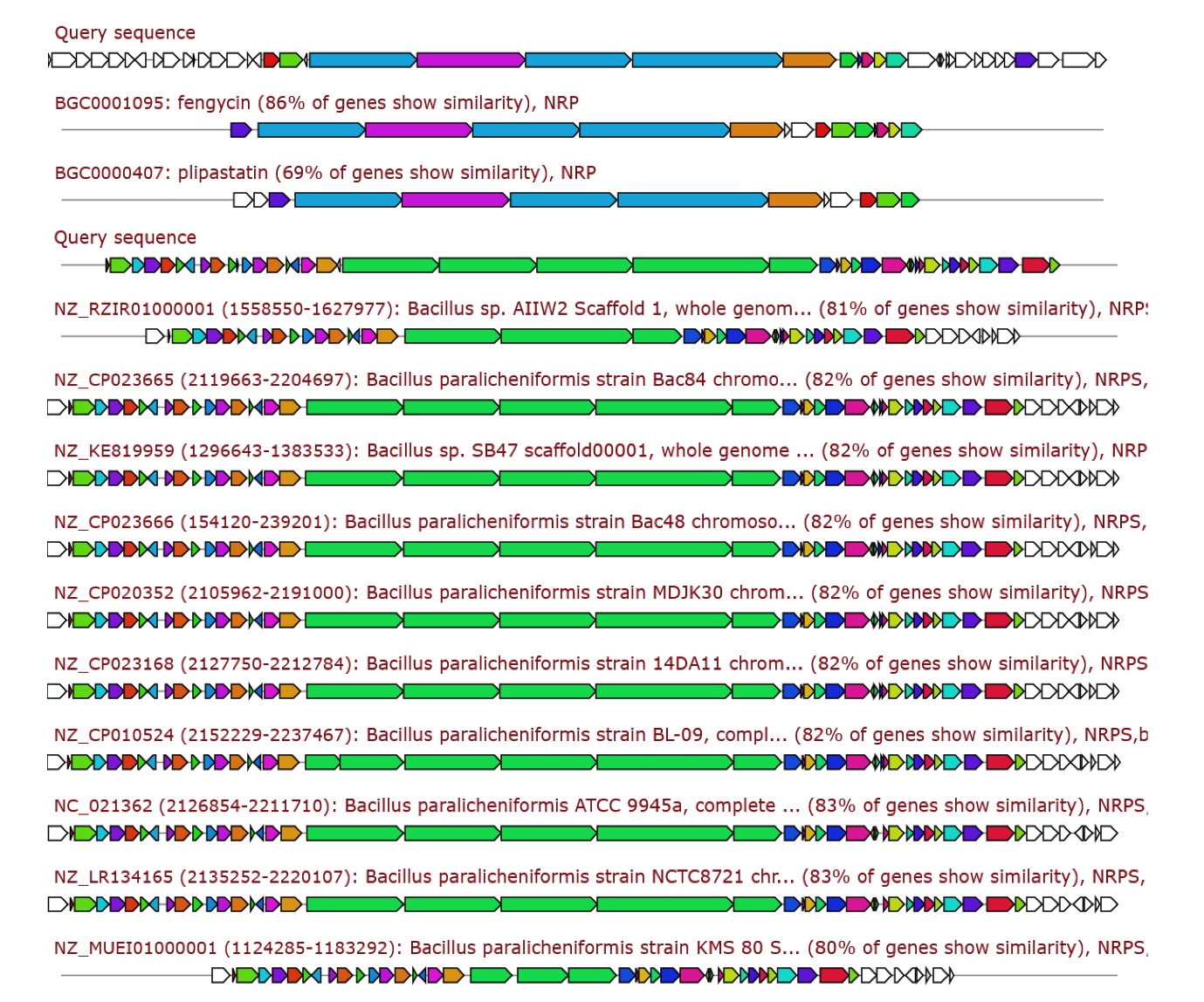


**Figure S4.** The Fengycin cluster blast of BP9 within *paralicheniformis* group of strains via Antismash V7.0beta1.


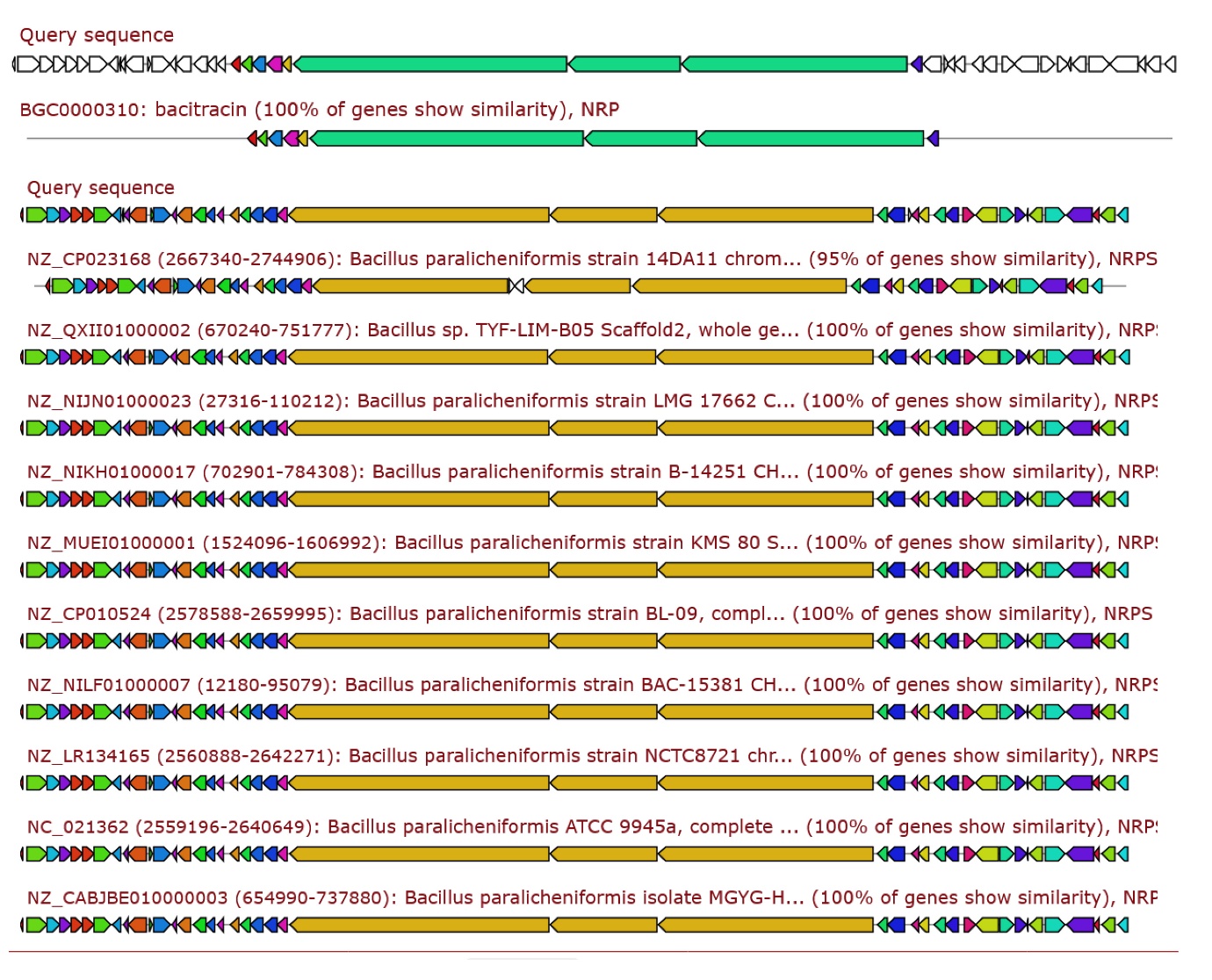


**Figure S5.** The Bacitracin cluster blast of BP9 within *paralicheniformis* group of strains via Antismash V7.0beta1.


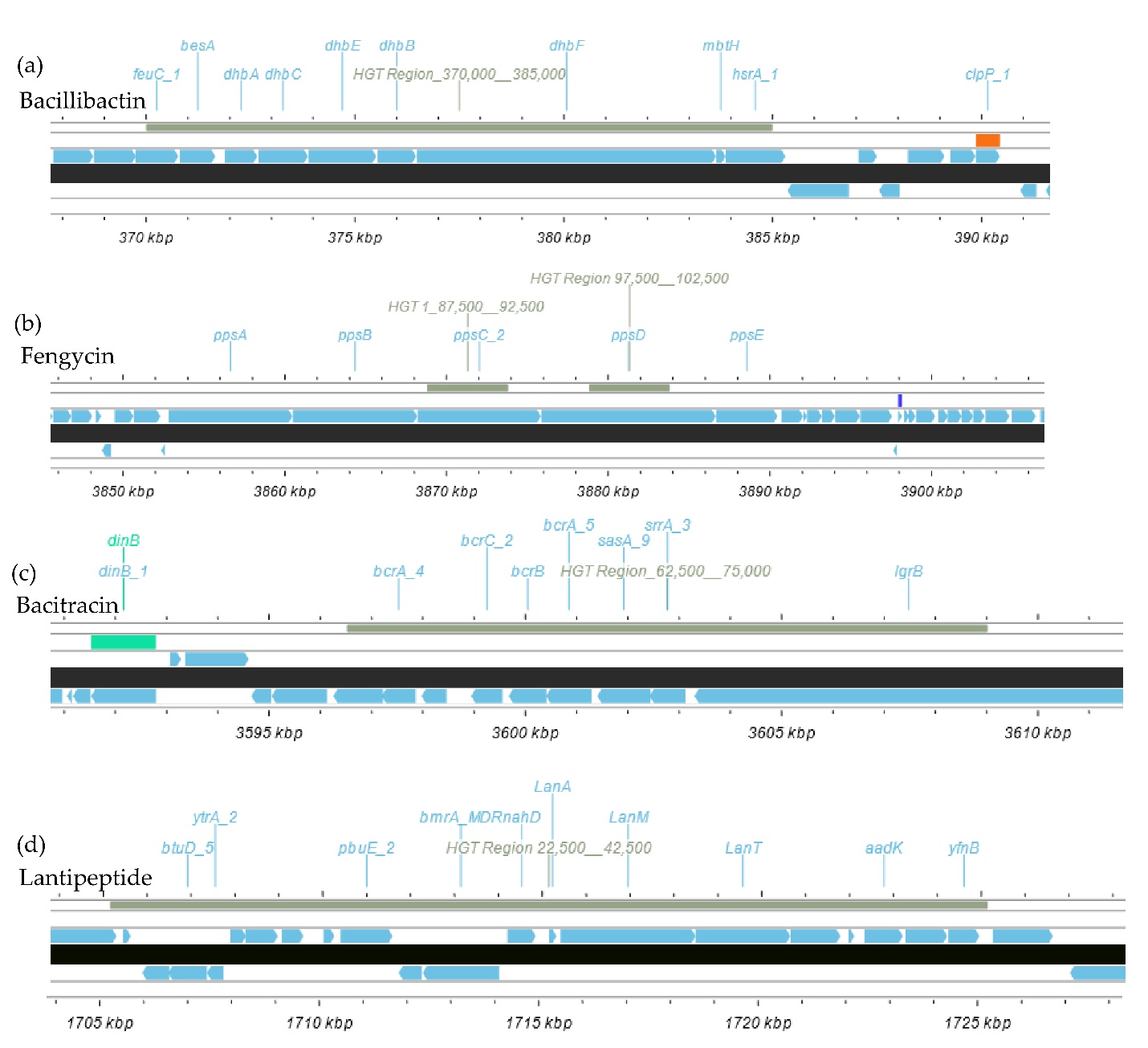
 **Figure S6.** The extended view of Horizontal gene transfer locations in secondary metabolites Bacilibactin, Fengycin, Bacitracin, and Lantipeptide in BP9.


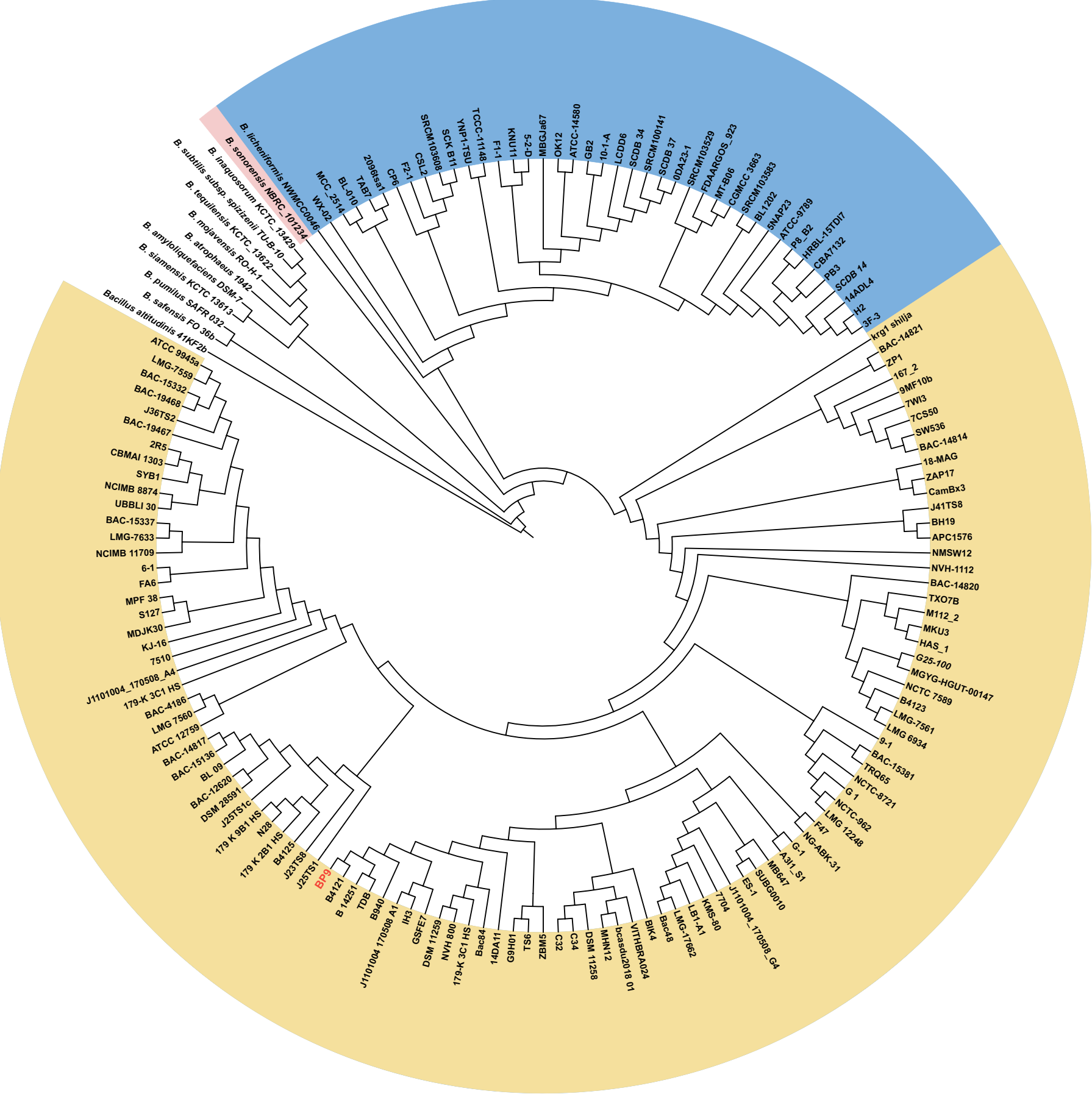


**Figure S7.** A phylogenetic tree constructed using 662 single copy core genes of 165 strains for the delineation of BP9. Blue color indicate the *B. licheniformis* strains and yellow color reflects the *B. paralicheniformis* strains, while BP9 is highlighted red.

**Table S1.** List of strains used in phylogenetic analysis of BP9 to construct Figure 4.

| *Paenibacillus plymyxa* | M1 | HE577054.1 |
| --- | --- | --- |
| *B. licheniformis* | 14ADL4 | CP026673.1 |
| *B. licheniformis* | CP6 | CP049330.1 |
| *B. licheniformis* | KNU11 | CP042252.1 |
| *B. licheniformis* | SCDB 34 | CP014793.1 |
| *B. licheniformis* | BL1202 | CP017247.1 |
| *B. licheniformis* | MT-B06 | CP032538.1 |
| *B.* para*licheniformis* | J41TS8 | AP025342.1 |
| *B.* para*licheniformis* | KJ-16 | LBMN02 |
| *B.* para*licheniformis* | FA6 | CP033198.1 |
| *B.* para*licheniformis* | Bac84 | CP023665.1 |
| *B.* para*licheniformis* | BP9 |  |
| *B.* para*licheniformis* | A4-3 | CP043501.1 |
| *B.* para*licheniformis* | 14DA11 | CP023168.1 |
| *B. sonorensis* | G25-136 | LDIP01 |
| *B. sonorensis* | J41TS2 | BORD01 |
| *B. sonorensis* | SRCM101395 | CP021920.1 |
| *B. velezensis* | FZB42 | CP000560.2 |
| *B. velezensis* | CBMB205 | CP014838.1 |
| B. siamensis | YB-1631 | CP110268.1 |
| B. siamensis | B28 | CP066219.1 |
| *B. atrophaeus* | GQJK17 | CP022653.1 |
| *B. atrophaeus* | BA59 | CP024051.1 |
| *B. subtilis* | NCIB3610 |  |
| *B. subtilis* | 168 | CP103783.1 |
| *B. mojavensis* | UCMB5075 | CP051464.1 |
| *B. mojavensis* | PS17 | CP066516.1 |
|  |  |  |

**Table S2.** List of strains used to construct a phylogenetic tree of BP9 with 165 strains and shown in Figure S8.

|  | Name | Strain | Acession ID |
| --- | --- | --- | --- |
| 1 | *B. altitudinis* | 41KF2b | ASJC00 |
| 2 | *B. amyloliquefaciens* | DSM-7 | FN597644 |
| 3 | *B. atrophaeus* | 1942 | CP002207 |
| 4 | *B. inaquosorum* | KCTC-13429 | AMXN00 |
| 5 | *B. mojavensis* | RO-H-1, KCTC 3706 | AFSI01 |
| 6 | *B. pumilus* | SAFR-032 | CP000813 |
| 7 | *B. safensis* | F0-36b | ASJD01 |
| 8 | *B. siamensis* | KCTC 13613 | AJVF01 |
| 9 | *B. sonorensis* | KCTC 13918 | AYTN01 |
| 10 | *B. subtilis subsp spizizenii* | TU-B-10 | CP002905 |
| 11 | *B. tequilensis* | KCTC 13622 | AYTO01 |
| 12 | *B. licheniformis* | 2096tsa1 | CP099860 |
| 13 | *B. licheniformis* | 3F-3 | JFYM01 |
| 14 | *B. licheniformis* | 5-2-D | AJLW01 |
| 15 | *B. licheniformis* | BL-010 | CP022477 |
| 16 | *B. licheniformis* | CGMCC-3663 | AMWQ01 |
| 17 | *B. licheniformis* | CSL2 | CP041154 |
| 18 | *B. licheniformis* | ATCC-14580 | CP000002 |
| 19 | *B. licheniformis* | F1-1 | AZSL01 |
| 20 | *B. licheniformis* | F2-1 | AJLW01 |
| 21 | *B.licheniformis* | LCDD6 | CP065029 |
| 22 | *B. licheniformis* | MBGJa67 | CP026522 |
| 23 | *B. licheniformis* | OK12 | CP060498 |
| 24 | *B. licheniformis* | WX-02 | CP012110 |
| 25 | *B. licheniformis* | MGYG-HGUT-02357 | LR698983 |
| 26 | *B. licheniformis* | 0DA23-1 | CP031126 |
| 27 | *B. licheniformis* | 14ADL4 | CP026673 |
| 28 | *B. licheniformis* | 5NAP23 | JYBQ00000000 |
| 29 | *B. licheniformis* | ATCC-9789 | CP023729 |
| 30 | *B. licheniformis* | BL1202 | CP017247 |
| 31 | *B. licheniformis* | CBA7132 | CP021970 |
| 32 | *B. licheniformis* | CP6 | CP049330 |
| 33 | *B. licheniformis* | FDAARGOS 923 | CP065647 |
| 34 | *B. licheniformis* | GB2 | JYGX00 |
| 35 | *B. licheniformis* | H2 | CP065943 |
| 36 | *B. licheniformis* | HRBL-15TDI7 | CP014781 |
| 37 | *B. licheniformis* | KNU11 | CP042252 |
| 38 | *B. licheniformis* | MCC-2514 | CP038186 |
| 39 | *B. licheniformis* | MT-B06 | CP032538 |
| 40 | *B. licheniformis* | NWMCC0046 | CP090312 |
| 41 | *B. licheniformis* | P8-B2 | CP045814 |
| 42 | *B. licheniformis* | PB3 | CP025226 |
| 43 | *B. licheniformis* | SCCB 37 | CP014794.1 |
| 44 | *B. licheniformis* | SCDB 14 | CP014842.1 |
| 45 | *B. licheniformis* | SCDB 34 | CP014793.1 |
| 46 | *B. licheniformis* | SCK B11 | CP014795 |
| 47 | *B. licheniformis* | SRCM100141 | CP021669.1 |
| 48 | *B. licheniformis* | SRCM103529 | CP035228.1 |
| 49 | *B. licheniformis* | SRCM103583 | CP035404.1 |
| 50 | *B. licheniformis* | SRCM103608 | CP035405.1 |
| 51 | *B. licheniformis* | TAB7 | CP027789 |
| 52 | *B. licheniformis* | TCCC-11148 | CP033218 |
| 53 | *B. licheniformis* | YNP1-TSU | MIGE000 |
| 55 | *B. paralichenformis* | BP9 | - |
| 56 | *B. paralicheniformis* | 14DA11 | CP023168 |
| 57 | *B. paralicheniformis* | 6 1 | NKBD01 |
| 58 | *B. paralicheniformis* | 9 1 | NKBC01 |
| 59 | *B. paralicheniformis* | A3I1_S1 | JAMBMA01 |
| 60 | *B. paralicheniformis* | ATCC 9945a | CP005965.1 |
| 61 | *B. paralicheniformis* | BIK4 | JAHWRD01 |
| 62 | *B. paralicheniformis* | BL-09 | NZ_CP010524.1 |
| 63 | *B. paralicheniformis* | Bac84 | NZ_CP023665 |
| 64 | *B. paralicheniformis* | ES 1 | NZ_CP083398 |
| 65 | *B. paralicheniformis* | FA6 | NZ_CP033198 |
| 66 | *B. paralicheniformis* | G1 | JABVEE01 |
| 67 | *B. paralicheniformis* | J1101004_170508_A1 | JADNAZ01 |
| 68 | *B. paralicheniformis* | J1101004_170508_A4 | JADMUO01 |
| 69 | *B. paralicheniformis* | J1101004_170508_G4 | JADNPI01 |
| 70 | *B. paralicheniformis* | J25TS1 | BOQZ01 |
| 71 | *B. paralicheniformis* | M112 2 | JAHTVI01 |
| 72 | *B. paralicheniformis* | MGYG HGUT 00147 | CABJBE01 |
| 73 | *B. paralicheniformis* | NCTC8721 | NZ_LR134165 |
| 74 | *B. paralicheniformis* | NG ABK 31 | NZ CP104093 |
| 75 | *B. paralicheniformis* | RSC 1 | NZ AP023088 |
| 76 | *B. paralicheniformis* | SUBG0010 | NZ CP068988 |
| 77 | *B. paralicheniformis* | TRQ65 | SAZD02000032 |
| 78 | *B. paralicheniformis* | ZP1 | JALIZJ010000054 |
| 79 | *B. paralicheniformis* | kGKOcqob9d bin 18 MAG | CAMIEI010000073 |
| 80 | *B. paralicheniformis* | 167/2 | MIZE01 |
| 81 | *B. paralicheniformis* | 179-I 1C1 HS | JAHHYC01 |
| 82 | *B. paralicheniformis* | 179-K 2B1 HS | JAHHXC01 |
| 83 | *B. paralicheniformis* | 179-K 3C1 HS | JAHHXA01 |
| 84 | *B. paralicheniformis* | 179-K 9B1 HS | JAHHXF01 |
| 85 | *B. paralicheniformis* | 2R5 | JAOWAK01 |
| 86 | *B. paralicheniformis* | 7510 | NPMO01 |
| 87 | *B. paralicheniformis* | 7704 | NPCO01 |
| 88 | *B. paralicheniformis* | 7CS50 | WLVZ01 |
| 89 | *B. paralicheniformis* | 7WI3 | WLVY01 |
| 90 | *B. paralicheniformis* | 9MF10b | WLVX01 |
| 91 | *B. paralicheniformis* | ATCC 12759 | JMPZ01 |
| 92 | *B. paralicheniformis* | B4121 | LKPO01 |
| 93 | *B. paralicheniformis* | B4123 | LKPP01 |
| 94 | *B. paralicheniformis* | B4125 | LKPR01 |
| 95 | *B. paralicheniformis* | B940 | JADRJL01 |
| 96 | *B. paralicheniformis* | BAC-12620 | NIND01 |
| 97 | *B. paralicheniformis* | BAC-14814 | NIMF01 |
| 98 | *B. paralicheniformis* | BAC-14817 | NIMC01 |
| 99 | *B. paralicheniformis* | BAC-14820 | NILZ01 |
| 100 | *B. paralicheniformis* | BAC-14821 | NILY01 |
| 101 | *B. paralicheniformis* | BAC-15136 | NILU01 |
| 102 | *B. paralicheniformis* | BAC-15332 | NILI01 |
| 103 | *B. paralicheniformis* | BAC-15337 | NILG01 |
| 104 | *B. paralicheniformis* | BAC-15381 | NILF01 |
| 105 | *B. paralicheniformis* | BAC-19467 | NIKZ01 |
| 106 | *B. paralicheniformis* | BAC-19468 | NIKY01 |
| 107 | *B. paralicheniformis* | BAC-4186 | NIJW01 |
| 108 | *B. paralicheniformis* | BH19 | PJMD01 |
| 109 | *B. paralicheniformis* | BL-09 | CP010524 |
| 110 | *B. paralicheniformis* | B-14251 | NIKH00 |
| 111 | *B. paralicheniformis* | Bac48 | NZ_CP023666 |
| 112 | *B. paralicheniformis* | C32 | RSBH01 |
| 113 | *B. paralicheniformis* | C34 | RSBG01 |
| 114 | *B. paralicheniformis* | CBMAI 1303 | CP033389.1 |
| 115 | *B. paralicheniformis* | CamBx3 | CP110812.1 |
| 116 | *B. paralicheniformis* | DSM 11258 | NIKU01 |
| 117 | *B. paralicheniformis* | DSM 11259 | NIKT01 |
| 118 | *B. paralicheniformis* | DSM 28591 | JANJZO01 |
| 119 | *B. paralicheniformis* | F47 | MYFI01 |
| 120 | *B. paralicheniformis* | G1 | NIJX01 |
| 121 | *B. paralicheniformis* | G25-100 | LDHI01 |
| 122 | *B. paralicheniformis* | G9H01 | JAIKUX01 |
| 123 | *B. paralicheniformis* | GSFE7 | JALJEF01 |
| 124 | *B. paralicheniformis* | HAS-1 | CP082896.1 |
| 125 | *B. paralicheniformis* | IH3 | JAJBZU01 |
| 126 | *B. paralicheniformis* | J23TS8 | BOQY01 |
| 127 | *B. paralicheniformis* | J25TS1 | BOQZ01 |
| 128 | *B. paralicheniformis* | J36TS2 | BORA01 |
| 130 | *B. paralicheniformis* | KJ-16 | LBMN02 |
| 131 | *B. paralicheniformis* | KMS 80 | MUEI01 |
| 132 | *B. paralicheniformis* | LB1-A1 | JALJCM01 |
| 133 | *B. paralicheniformis* | LMG 12248 | NIMX01 |
| 134 | *B. paralicheniformis* | LMG 17662 | NIJN01 |
| 135 | *B. paralicheniformis* | LMG 6934 | NIJV01 |
| 136 | *B. paralicheniformis* | LMG 7559 | NIJT01 |
| 137 | *B. paralicheniformis* | LMG 7560 | NIJS01 |
| 138 | *B. paralicheniformis* | LMG 7561 | NIJR01 |
| 139 | *B. paralicheniformis* | LMG 7633 | NIMV01 |
| 140 | *B. paralicheniformis* | MB647 | JABWTN01 |
| 141 | *B. paralicheniformis* | MDJK30 | CP020352.1 |
| 142 | *B. paralicheniformis* | MHN12 | JANHBI01 |
| 143 | *B. paralicheniformis* | MKU3 | MSZY00 |
| 144 | *B. paralicheniformis* | MPF 38 | JAMAWU01 |
| 145 | *B. paralicheniformis* | N28 | JAHLOI01 |
| 146 | *B. paralicheniformis* | NCIMB 11709 | NINC01 |
| 147 | *B. paralicheniformis* | NCIMB 8874 | MBGK01 |
| 148 | *B. paralicheniformis* | NCTC 7589 | NJHC01 |
| 149 | *B. paralicheniformis* | NCTC 962 | NJHA01 |
| 150 | *B. paralicheniformis* | NMSW12 | PVQR01 |
| 151 | *B. paralicheniformis* | NVH 1112 | NIKL01 |
| 152 | *B. paralicheniformis* | NVH 800 | NIKK01 |
| 154 | *B. paralicheniformis* | S127 | LFIM01 |
| 155 | *B. paralicheniformis* | SW536 | SJOW00 |
| 156 | *B. paralicheniformis* | SYB1 | JANKJO01 |
| 157 | *B. paralicheniformis* | TDB | JAOPJE01 |
| 158 | *B. paralicheniformis* | TS6 | SHMY00 |
| 159 | *B. paralicheniformis* | TXO7B 1SG6 | CP082897 |
| 160 | *B. paralicheniformis* | UBBLI 30 | SULF00 |
| 161 | *B. paralicheniformis* | VITHBRA024 | NZ CP084682 |
| 162 | *B. paralicheniformis* | ZAP17 | NZ CP049698 |
| 163 | *B. paralicheniformis* | ZBM5 J | AIFOO01 |
| 164 | *B. paralicheniformis* | bcasdu2018 01 | JAGTPZ01 |
| 165 | *B. paralicheniformis* | krg1 shilja | WHJA01 |

**Table S3.** List of strains used in Pan Genome analysis

|  | *Bacillus paralicheniformis* | NCBI accession ID |
| --- | --- | --- |
| 1 | Bac84 | CP023665.1 |
| 2 | A4-3 | CP043501.1 |
| 3 | 14DA11 | CP023168.1 |
| 4 | J41TS8 | AP025342.1 |
| 5 | CBMAI 1303 | CP033389.1 |
| 6 | Bac48 | CP023666.1 |
| 7 | CamBx3 | CP110812.1 |
| 8 | FA6 | CP033198.1 |
| 9 | NCTC8721 | LR134165.1 |
| 10 | J36TS2 | AP025340.1 |
| 11 | J25TS1 | AP025339.1 |
| 12 | BL-09 | CP010524.1 |
| 13 | MDJK30 | CP020352.1 |
| 14 | RSC-1 | AP023088.1 |
| 15 | RSC-2 | AP023089.1 |
| 16 | SUBG0010 | CP068988.1 |
| 17 | ATCC 9945a | CP005965.1 |
| 18 | ES-1 | CP083398.1 |
| 19 | NG-ABK-31 | CP104093.1 |
| 20 | VITHBRA024 | CP084682.1 |
| 21 | ZAP17 | CP049698.1 |
| 22 | KJ-16 | LBMN02 |
| 23 | J23TS8 | BOQY01 |
| 24 | 179-K 2B1 HS | JAHHXC01 |
| 25 | J25TS1 | BOQZ01 |
| 26 | 179-K 9B1 HS | JAHHXF01 |
| 27 | ZP1 | JALIZJ01 |
| 28 | N28 | JAHLOI01 |
| 29 | MPF 38 | JAMAWU01 |
| 30 | MB647 | JABWTN01 |
| 31 | G1 | JABVEE01 |
| 32 | BAC-12620 | NIND01 |
| 33 | ZBM5 | JAIFOO01 |
| 34 | KMS 80 | MUEI01 |
| 35 | B-14251 | NIKH01 |
| 36 | 7WI3 | WLVY01 |
| 37 | 9MF010a | WLVW01 |
| 38 | 9MF10b | WLVX01 |
| 39 | 7CS50 | WLVZ01 |
| 40 | B4125 | LKPR01 |

**Table S4.** List of mobile elements (phages, transferases, transposases, conjugative elements) Ribosomal Binding Motif, and their GC content

| Gene name | Major category | RBS Motif | Specific Contig |
| --- | --- | --- | --- |
| Ung | Replication/recombination/repair | AGGAG | BP91 |
| Clpp | Phage | AGGAG | BP91 |
| Ssb | Replication/recombination/repair | GGAGG | BP91 |
| - | Integration/excision | GGAGG | BP91 |
| - | Integration/excision | None | BP9_2 |
| Oppf | Transfer | AGGAGG | BP9_2 |
| Oppd | Transfer | AGGAG | BP9_2 |
| Oppf | Transfer | AGxAGG/AGGxGG | BP9_2 |
| Nfua | Phage | None | BP9_3 |
| Rnr | Replication/recombination/repair | None | BP9_3 |
| Dps | Replication/recombination/repair | AGGAGG | BP9_4 |
| Pola | Replication/recombination/repair | AGGAGG | BP9_4 |
| Mutm | Replication/recombination/repair | AGGA | BP9_4 |
| Uvrc | Replication/recombination/repair | AGGA | BP9_4 |
| Rph | Replication/recombination/repair | GGAGG | BP9_4 |
| Rdgb | Replication/recombination/repair | GGA/GAG/AGG | BP9_4 |
| Clpx | Phage | GGA/GAG/AGG | BP9_4 |
| - | Integration/excision | GGAGG | BP9_5 |
| Ruvb | Replication/recombination/repair | GGA/GAG/AGG | BP9_5 |
| Rara | Replication/recombination/repair | GGAG/GAGG | BP9_5 |
| Oppf | Transfer | GGA/GAG/AGG | BP9_5 |
| Oppd | Transfer | AGGA | BP9_5 |
| Comeb | Transfer | GGAGG | BP9_5 |
| Dnak | Phage | AGGAGG | BP9_5 |
| Dnaj | Replication/recombination/repair | GGAG/GAGG | BP9_5 |
| Nfo | Replication/recombination/repair | AGxAGG/AGGxGG | BP9_5 |
| Liga | Replication/recombination/repair | AGGA | BP9_7 |
| Pcra | Replication/recombination/repair | AGxAGG/AGGxGG | BP9_7 |
| - | Transfer | AGxAGG/AGGxGG | BP9_7 |
| Parc | Replication/recombination/repair | AGGAGG | BP9_8 |
| Gyrb | Replication/recombination/repair | GGA/GAG/AGG | BP9_8 |
| - | Integration/excision | GGAG/GAGG | BP9_8 |
| Lexa | Phage | GGAG/GAGG | BP9_8 |
| - | Transfer | AGGAGG | BP9_8 |
| Hfq | Phage | AGGAGG | BP9_8 |
| Muts | Replication/recombination/repair | GGAG/GAGG | BP9_8 |
| Reca | Replication/recombination/repair | AGGAGG | BP9_8 |
| Ndoa_1 | Stability/transfer/defense | GGAGG | BP9_9 |
| - | Stability/transfer/defense | GGA/GAG/AGG | BP9_9 |
| Topb | Replication/recombination/repair | None | BP9_9 |
| - | Integration/excision | GGA/GAG/AGG | BP9_9 |
| Tnp_2 | Integration/excision | None | BP9_10 |
| Oppf | Transfer | GGA/GAG/AGG | BP9_10 |
| Oppd | Transfer | AGGAG | BP9_10 |
| Xhlb | Phage | GGAGG | BP9_10 |
| Xhla | Phage | GGAG/GAGG | BP9_10 |
| Xkdu | Phage | GGA/GAG/AGG | BP9_10 |
| Xkdt | Phage | AGGAG/GGAGG | BP9_10 |
| - | Phage | AGGAG | BP9_10 |
| - | Phage | AGxAGG/AGGxGG | BP9_10 |
| - | Phage | AGGA | BP9_10 |
| - | Phage | AGGAG | BP9_10 |
| Xkdm | Phage | GGAGG | BP9_10 |
| Xkdk | Phage | AGGAGG | BP9_10 |
| - | Phage | GGAGG | BP9_10 |
| - | Phage | GGAG/GAGG | BP9_10 |
| Xtmb | Phage | GGA/GAG/AGG | BP9_10 |
| Xtma | Phage | AGxAGG/AGGxGG | BP9_10 |
| Clpp | Phage | AGGAGG | BP9_11 |
| Uvra | Replication/recombination/repair | AGGA | BP9_11 |
| Uvrb | Replication/recombination/repair | GGAGG | BP9_11 |
| Rnj | Replication/recombination/repair | GGAG/GAGG | BP9_12 |
| Abrb | Transfer | AGxAGG/AGGxGG | BP9_12 |
| - | Transfer | GGAG/GAGG | BP9_12 |
| Cina | Transfer | GGA/GAG/AGG | BP9_13 |
| Nusa | Phage | AGxAGG/AGGxGG | BP9_13 |
| Polc | Replication/recombination/repair | GGAGG | BP9_13 |
| Topa | Replication/recombination/repair | None | BP9_13 |
| Recg | Replication/recombination/repair | AGGAG/GGAGG | BP9_13 |
| Hup | Replication/recombination/repair | AGGAGG | BP9_15 |
| Nth | Replication/recombination/repair | GGA/GAG/AGG | BP9_15 |
| Rlml | Stability/transfer/defense | GGA/GAG/AGG | BP9_15 |
| - | Integration/excision | GGAGG | BP9_15 |
| Gyra | Replication/recombination/repair | GGAGG | BP9_16 |
| Gyrb | Replication/recombination/repair | - | BP9_16 |
| Recf | Replication/recombination/repair | AGxAGG/AGGxGG | BP9_16 |
| Dnaa | Replication/recombination/repair | GGA/GAG/AGG | BP9_16 |
| Soj_2 | Replication/recombination/repair | GGA/GAG/AGG | BP9_16 |
| Parb | Replication/recombination/repair | AGGA/GGAG/GAGG | BP9_16 |
| Ssb | Replication/recombination/repair | AGGA/GGAG/GAGG | BP9_16 |
| Dnab | Replication/recombination/repair | AGGAG/GGAGG | BP9_16 |
| Dinb | Replication/recombination/repair | GGAGG | BP9_17 |
| - | Integration/excision | GGAG/GAGG | BP9_19 |
| Nusg | Phage | GGA/GAG/AGG | BP9_21 |
| Sigh | Integration/excision | GGA/GAG/AGG | BP9_21 |
| Rada | Replication/recombination/repair | GGA/GAG/AGG | BP9_21 |
| Clpb | Phage | AGGAGG | BP9_21 |
| Ftsh | Phage | AGGAGG | BP9_22 |
| Abrb | Transfer | GGAGG | BP9_22 |
| Ftsz | Replication/recombination/repair | None | BP9_23 |
| Recr | Replication/recombination/repair | AGGAGG | BP9_26 |
| Groel | Phage | AGGAGG | BP9_27 |
| Gros | Phage | AGGAGG | BP9_27 |
| Rex | Stability/transfer/defense | GGAGG | BP9_27 |
| - | Integration/excision | None | BP9_42 |
| - | Integration/excision | None | BP9_52 |

**Table S5.** Multi Locus Sequence Analysis (MLSA) of BP9 genome containing multiple sets of alleles that could be used as genetic markers to distinguish the BP9 from other strains.

| Locus | Allele | Length | Contig | Start position | End position |
| --- | --- | --- | --- | --- | --- |
| *adk* | 3 | 465 | BP9_21 | 15801 | 16265 |
| *ccpA* | 2 | 561 | BP9_4 | 117642 | 118202 |
| *recF* | 5 | 561 | BP9_16 | 5858 | 6418 |
| *rpoB* | 3 | 495 | BP9_21 | 38842 | 39336 |
| *spo0A* | 9 | 558 | BP9_18 | 82600 | 83157 |
| *sucC* | 8 | 549 | BP9_13 | 89863 | 90411 |

**Table S6.** 16S rDNA blast analysis by type genome server and gctype pipeline.

| 16S rDNA of BP9 | Identity |
| --- | --- |
| *B. paralicheniformis (KJ-16)* (LBMN02) | **100.0** |
| *B. haynesii* NRRL B-41327 | 99.469 |
| *B. sonorensis* KCTC 13918 | 99.219 |
| *B. licheniformis* DSM-13 | 99.155 |
| *B. subtilis subsp. inaquosorum* KCTC 13429 | 98.764 |
| *B. subtilis subsp. spizizenii* TU-B-10 | 98.764 |

**Table S7.** Digital DNA-DNA Hybridization and Average Nucleotide Identity (ANI) values calculated by GGDC, TYGS server using BP9 genome as query

| Query strain | Subject strain | | dDDH (d4, in %) | | C.I. (d4, in %) | | G+C content difference (in %) | | | |  |
| --- | --- | --- | --- | --- | --- | --- | --- | --- | --- | --- | --- |
| BP9 | ***B. paralicheniformis* KJ-16** | | **92.6** | | [90.6 - 94.2] | | | 1.2 | | |  |
| BP9 | ***B. licheniformis* ATCC 14580** | | **57.9** | | [55.1 - 60.7] | | | 0.76 | | |  |
| BP9 | *B. sonorensis* KCTC 13918 | | 31.6 | | [29.2 - 34.1] | | | 1.43 | | |  |
| Species | | **Strain name** | | **G2G dif*** | | **ANIb** | | | **FastANI** | **OrthoANIu** | |
| *B. licheniformis* | | **ATCC 14580** | | **0.0407** | | **95.12** | | | **94.99** | **95.15** | |
| *B. paralicheniformis* (LBMN02) | | **KJ-16** | | **0.0092** | | **98.99** | | | **98.91** | **99.02** | |
| *B. sonorensis* (AYTN01) | | KCTC 13918 | | 0.142 | | 80.91 | | | 82.82 | 81.37 | |
| *B. tequilensis* (UAQB01) | | NCTC13306 | | 0.1973 | | 71.65 | | | 78.7 | 72.67 | |
| *B. mojavensis* (AYTL01) | | KCTC 3706(T) | | 0.2036 | | 71.63 | | | 78.55 | 72.8 | |
| *B. velezensis* (CP011937.1) | | CBMB205 | | 0.2036 | | 71.67 | | | 78.73 | 72.53 | |
| *B. subtilis subsp. Stercoris* (JHCA01) | | D7XPN1 | | 0.2036 | | 71.65 | | | 78.65 | 72.9 | |

*** G2G dif= Genome to Genome difference**

**Table S8.** Identification of biosynthetic gene clusters (BGCs) in BP9 genome via Antismash analysis

| Cluster | Type | From | To | Most similar known cluster | |  |
| --- | --- | --- | --- | --- | --- | --- |
|  |  |  |  | **Name** | **Similarity** | **MIBiG** |
| 1 | Lassopeptide | 231,145 | 253,606 | - | - | BGC0002325 |
| 2 | NRPS | 351,891 | 403,646 | Bacillibactin | 100% | BGC0002695 |
| 3 | Thiopeptide, RiPP- | 108,653 | 149,857 | Butirosin A/B | 7% | BGC0001638 |
| 4 | NRPS-indepedent Siderophore | 267,812 | 283,275 | schizokinen | 60% | BGC0002683 |
| 5 | CDPS | 280183 | 300932 | Pulcherriminic | 66% | BGC0002633 |
| 6 | Lanthipeptide class ii | 22,766 | 45,840 | Geobacillin II | 50% | BGC0002103 |
| 7 | NRPS | 200,908 | 244,066 | Lichenysin/NRP | 64% | BGC0000516 |
| 8 | terpene | 44,552 | 66,441 | - | - | BGC0002173 |
| 9 | T3PKS | 84,414 | 116,659 | - | - | BGC0000282 |
| 10 | NRPS | 49,280 | 132,171 | Bacitracin/ NRP | 100% | BGC0000310 |
| 11 | RiPP-Like | 10,644 | 20,988 | - | - |  |
| 12 | NRPS/Betalactone | 51,493 | 129,160 | Fengysin/NRP | 86% | BGC0000426. |

**Table S9.** PIFAR is an open-access, web-based tool to identify plant-bacteria interactions

| Category | Description /gene name | No. |
| --- | --- | --- |
| Antibiotic | Bacillibactin | 4 |
|  | Lanthipeptide/ Butirosin class II | 4 |
|  | Bacitracin | 4 |
|  | Fengysin | 2 |
| Biofilm | TasA anchoring/assembly protein | 6 |
|  | Major biofilm matrix component | 1 |
|  | Hyp1 | 1 |
|  | Biofilm-surface layer protein A | 2 |
| Resistance or  Detoxification | Cobalt-zinc-cadmium resistance | 8 |
|  | Resistance for penicillin | 1 |
|  | Resistance to fluoroquinolones | 1 |
|  | Fosfomycin resistance | 2 |
|  | Beta-lactamase | 1 |
|  | Resistance to chromium compounds | 2 |
|  | Multidrug Resistance Efflux Pumps | 2 |
|  | isothiocyanate resistance | 5 |
|  | sapABCDF | 1 |
|  | katB | 1 |
|  | dps | 5 |
| EPSs | Copper homeostasis | 1 |
|  | EpsA, CpsA | 1 |
| MDRs | lytR | 2 |
|  | ACR tran | 22 |
|  | Multi Drug Res | 4 |
| PCWDEs | lipA | 1 |
|  | pectin methylesterase | 1 |
|  | peh-1 | 1 |
|  | Cellulase | 1 |
| Proteases | Pec lyase C | 2 |
| Volatiles | htrA/B | 6 |
|  | Acetoin bdhA-2 | 1 |
| Siderophores | arthrobactin/ Anthrachelin | 4 |
|  | 2,3-butanediol | 4 |
|  | dhb | 2 |
| Metabolism | tryptophan/ trpCG | 2 |
|  | glutamate synthase gltBD | 1 |
|  | aroK | 3 |
|  | aroQ | 2 |
|  | asnB | 1 |
|  | aroC | 1 |
|  | purine biosynthesis purD | 1 |
| LPSs | GlgD | 1 |
| Hormones | Auxin | 1 |
| MAMPs | Teichuronic acid | 6 |
|  | Chemotaxis protein *cheA* | 1 |
|  | Chemotaxis protein *cheW* | 1 |
|  | Chemotaxis protein *cheY* | 1 |
|  | Chemoreceptor glutamine deamidase *cheD* | 1 |
|  | Chemotaxis response regulator  protein-glutamate methylesterase *cheB* | 1 |
|  | CheY-P phosphatase *cheC* | 1 |
|  | YvyG | 1 |
|  | Methyl-accepting chemotaxis protein McpC/A | 2 |
|  | Chemotaxis protein *PomA* | 1 |

**Table S10.** The identified antibiotic resistance genes in BP9

| Resistance Type | Description | Resistance Profile | Require | Protein |
| --- | --- | --- | --- | --- |
| *baca*  *bcra*  bcrc | Undecaprenyl pyrophosphate phosphatase, which consists in the sequestration of Undecaprenyl pyrophosphate. | bacitracin | *bacA* | BP9_00729  BP9_00823 |
|  | ABC transporter system, bacitracin efflux pump. | bacitracin | *bcrC* | BP9_03643 |
|  | ABC transporter system, bacitracin efflux pump. | bacitracin | *bcrA* | BP9_03641 |
| bl2a_iii2 | Class A beta-lactamase. This enzyme breaks the beta-lactam antibiotic ring open and deactivates the molecule's antibacterial properites. | penicillin |  | BP9_01808 |
| *ermd* | rRNA adenine N-6-methyltransferase, which can methylate adenine at position 2058 of 23S rRNA, conferring resistance to erythromycin. | streptogramin_b, lincosamide, macrolide | | BP9_01570 |
| *fosb* | Glutathione transferase, metalloglutathione transferase which confers resistance to fosfomycin by catalyzing the addition of glutathione to fosfomycin | fosfomycin |  | BP9_02514 |

**Table S11.** The HGT genes identified in BP9 and their location at genome

| Contig | Start | Stop | Score | Threhold |
| --- | --- | --- | --- | --- |
| BP9_4 | 1 | 7500 | 0.66 | 26.198 |
| BP9_4 | 25000 | 30000 | 0.295 | 26.198 |
| BP9_4 | 35000 | 42500 | 0.332 | 26.198 |
| BP9_4 | 75000 | 85000 | 0.388 | 26.198 |
| BP9_4 | 90000 | 95000 | 0.289 | 26.198 |
| BP9_4 | 112500 | 117500 | 0.345 | 26.198 |
| BP9_4 | 125000 | 132500 | 0.488 | 26.198 |
| BP9_4 | 167500 | 177500 | 0.405 | 26.198 |
| BP9_4 | 215000 | 222500 | 0.351 | 26.198 |
| BP9_4 | 225000 | 232500 | 0.322 | 26.198 |
| BP9_4 | 255000 | 260000 | 0.344 | 26.198 |
| BP9_4 | 302500 | 307500 | 0.323 | 26.198 |
| BP9_4 | 315138 | 320138 | 0.503 | 26.198 |
| BP9_5 | 72500 | 77500 | 0.299 | 27.469 |
| BP9_5 | 115000 | 125000 | 0.339 | 27.469 |
| BP9_5 | 130000 | 135000 | 0.313 | 27.469 |
| BP9_5 | 137500 | 147500 | 0.357 | 27.469 |
| BP9_5 | 150000 | 172500 | 0.535 | 27.469 |
| BP9_5 | 200000 | 207500 | 0.359 | 27.469 |
| BP9_5 | 230000 | 237500 | 0.702 | 27.469 |
| BP9_8 | 5000 | 12500 | 0.428 | 30.393 |
| BP9_8 | 32500 | 37500 | 0.364 | 30.393 |
| BP9_8 | 42500 | 50000 | 0.355 | 30.393 |
| BP9_8 | 92500 | 105000 | 0.564 | 30.393 |
| BP9_8 | 112500 | 117500 | 0.422 | 30.393 |
| BP9_8 | 120000 | 135000 | 0.638 | 30.393 |
| BP9_8 | 172500 | 177500 | 0.501 | 30.393 |
| BP9_9 | 27500 | 55000 | 0.588 | 23.451 |
| BP9_10 | 1 | 5000 | 0.519 | 29.935 |
| BP9_10 | 22500 | 27500 | 0.344 | 29.935 |
| BP9_10 | 35000 | 40000 | 0.309 | 29.935 |
| BP9_10 | 72500 | 85000 | 0.332 | 29.935 |
| BP9_10 | 110000 | 115000 | 0.403 | 29.935 |
| BP9_10 | 117500 | 125000 | 0.515 | 29.935 |
| BP9_10 | 135000 | 142500 | 0.467 | 29.935 |
| BP9_10 | 150000 | 157500 | 0.823 | 29.935 |
| BP9_10 | 171289 | 176289 | 0.558 | 29.935 |
| BP9_11 | 117500 | 122500 | 0.171 | 15.093 |
| BP9_11 | 152500 | 167500 | 0.537 | 15.093 |
| BP9_12 | 45000 | 52500 | 0.325 | 23.752 |
| BP9_12 | 130000 | 145000 | 0.608 | 23.752 |
| BP9_13 | 20000 | 40000 | 0.681 | 39.998 |
| BP9_13 | 67500 | 82500 | 0.493 | 39.998 |
| BP9_13 | 110000 | 115000 | 0.461 | 39.998 |
| BP9_14 | 20000 | 35000 | 0.726 | 36.433 |
| BP9_14 | 42500 | 50000 | 0.94 | 36.433 |
| BP9_14 | 92500 | 97500 | 0.416 | 36.433 |
| BP9_14 | 117500 | 122500 | 0.422 | 36.433 |
| BP9_15 | 5000 | 12500 | 0.937 | 36.71 |
| BP9_15 | 47500 | 57500 | 0.393 | 36.71 |
| BP9_15 | 92500 | 100000 | 0.776 | 36.71 |
| BP9_15 | 111659 | 116659 | 0.468 | 36.71 |
| BP9_17 | 7500 | 20000 | 0.713 | 42.928 |
| BP9_17 | 25000 | 30000 | 0.616 | 42.928 |
| BP9_17 | 37500 | 42500 | 0.445 | 42.928 |
| BP9_17 | 50000 | 55000 | 0.568 | 42.928 |
| BP9_17 | 62500 | 75000 | 0.527 | 42.928 |
| BP9_17 | 97500 | 102500 | 0.545 | 42.928 |
| BP9_17 | 105000 | 117500 | 0.653 | 42.928 |
| BP9_17 | 142500 | 147500 | 0.456 | 42.928 |
| BP9_17 | 150000 | 157500 | 0.618 | 42.928 |
| BP9_18 | 12500 | 22500 | 0.503 | 16.984 |
| BP9_19 | 87500 | 92500 | 0.336 | 32.367 |
| BP9_19 | 97500 | 102500 | 0.454 | 32.367 |
| BP9_19 | 132500 | 154968 | 0.704 | 32.367 |
| BP9_20 | 1 | 10000 | 0.621 | 35.607 |
| BP9_20 | 45000 | 52500 | 0.412 | 35.607 |
| BP9_20 | 61642 | 66642 | 0.459 | 35.607 |
| BP9_21 | 17500 | 37500 | 0.708 | 53.363 |
| BP9_21 | 45000 | 60384 | 0.571 | 53.363 |
| BP9_22 | 10000 | 20000 | 0.906 | 55.437 |
| BP9_22 | 22500 | 35000 | 0.666 | 55.437 |
| BP9_22 | 51350 | 56350 | 0.7 | 55.437 |
| BP9_23 | 12500 | 20000 | 0.791 | 50.512 |
| BP9_23 | 25000 | 30000 | 1 | 50.512 |
| BP9_23 | 37500 | 42500 | 0.698 | 50.512 |
| BP9_24 | 1 | 5000 | 1 | 49.253 |
| BP9_24 | 22500 | 27500 | 0.894 | 49.253 |
| BP9_24 | 34013 | 39013 | 0.636 | 49.253 |
| BP9_26 | 7500 | 17500 | 0.954 | 82.403 |
